# Comprehensive Analysis of Transcriptome Variation Uncovers Known and Novel Driver Events in T-Cell Acute Lymphoblastic Leukemia

**DOI:** 10.1371/journal.pgen.1003997

**Published:** 2013-12-19

**Authors:** Zeynep Kalender Atak, Valentina Gianfelici, Gert Hulselmans, Kim De Keersmaecker, Arun George Devasia, Ellen Geerdens, Nicole Mentens, Sabina Chiaretti, Kaat Durinck, Anne Uyttebroeck, Peter Vandenberghe, Iwona Wlodarska, Jacqueline Cloos, Robin Foà, Frank Speleman, Jan Cools, Stein Aerts

**Affiliations:** 1 Laboratory of Computational Biology, Center for Human Genetics, KU Leuven, Leuven, Belgium; 2 Laboratory for the Molecular Biology of Leukemia, Center for Human Genetics, KU Leuven and Center for the Biology of Disease, VIB, Leuven, Belgium; 3 Division of Hematology, Department of Cellular Biotechnologies and Hematology, ‘Sapienza’ University of Rome, Rome, Italy; 4 Center for Medical Genetics, Ghent University, Ghent, Belgium; 5 Pediatric Hemato-Oncology, University Hospitals Leuven, Leuven, Belgium; 6 Pediatric Oncology/Hematology and Hematology, VU Medical Center, Amsterdam, The Netherlands; Centre for Cancer Biology, SA Pathology, Australia

## Abstract

RNA-seq is a promising technology to re-sequence protein coding genes for the identification of single nucleotide variants (SNV), while simultaneously obtaining information on structural variations and gene expression perturbations. We asked whether RNA-seq is suitable for the detection of driver mutations in T-cell acute lymphoblastic leukemia (T-ALL). These leukemias are caused by a combination of gene fusions, over-expression of transcription factors and cooperative point mutations in oncogenes and tumor suppressor genes. We analyzed 31 T-ALL patient samples and 18 T-ALL cell lines by high-coverage paired-end RNA-seq. First, we optimized the detection of SNVs in RNA-seq data by comparing the results with exome re-sequencing data. We identified known driver genes with recurrent protein altering variations, as well as several new candidates including *H3F3A, PTK2B*, and *STAT5B*. Next, we determined accurate gene expression levels from the RNA-seq data through normalizations and batch effect removal, and used these to classify patients into T-ALL subtypes. Finally, we detected gene fusions, of which several can explain the over-expression of key driver genes such as *TLX1, PLAG1, LMO1*, or *NKX2-1*; and others result in novel fusion transcripts encoding activated kinases (*SSBP2-FER* and *TPM3-JAK2*) or involving *MLLT10*. In conclusion, we present novel analysis pipelines for variant calling, variant filtering, and expression normalization on RNA-seq data, and successfully applied these for the detection of translocations, point mutations, INDELs, exon-skipping events, and expression perturbations in T-ALL.

## Author Summary

The quest for somatic mutations underlying oncogenic processes is a central theme in today's cancer research. High-throughput genomics approaches including amplicon re-sequencing, exome re-sequencing, full genome re-sequencing, and SNP arrays have contributed to cataloguing driver genes across cancer types. Thus far transcriptome sequencing by RNA-seq has been mainly used for the detection of fusion genes, while few studies have assessed its value for the combined detection of SNPs, INDELs, fusions, gene expression changes, and alternative transcript events. Here we apply RNA-seq to 49 T-ALL samples and perform a critical assessment of the bioinformatics pipelines and filters to identify each type of aberration. By comparing to exome re-sequencing, and by exploiting the catalogues of known cancer drivers, we identified many known and several novel driver genes in T-ALL. We also determined an optimal normalization strategy to obtain accurate gene expression levels and used these to identify over-expressed transcription factors that characterize different T-ALL subtypes. Finally, by PCR, cloning, and *in vitro* cellular assays we uncover new fusion genes that have consequences at the level of gene expression, oncogenic chimaeras, and tumor suppressor inactivation. In conclusion, we present the first RNA-seq data set across T-ALL patients and identify new driver events.

## Introduction

T-cell acute lymphoblastic leukemia (T-ALL) is an aggressive malignancy that accounts for approximately 15% of pediatric and 25% of adult ALL cases. Despite improved outcome over the years, about 25% of children and 50% of adults still fail to respond to intensive chemotherapy protocols or relapse [1]. Improved understanding of T-ALL biology through the identification and characterization of oncogenic lesions is expected to lead to a better prognostic classification and the development of new targeted therapeutic strategies.

T-ALL is caused by the accumulation of multiple oncogenic mutations that have been identified through characterization of chromosomal aberrations and candidate gene sequencing [2]. Chromosomal translocations in T-ALL frequently involve the T-cell receptor (*TCR*) loci, whereby *TCR* regulatory elements become juxtaposed to genes that are normally not expressed in T-cells [3], [4]. In this way, a specific set of recurrently over-expressed transcription factors (TFs) have been documented, including *TLX1, TLX3, TAL1, LMO1, HOXA*, and *NKX* family members [5]. T-ALL samples expressing each of these transcription factors show a distinctive gene expression signature and as such these transcription factors define distinct molecular subtypes in T-ALL [6]. Chromosomal rearrangements can also lead to large chromosomal deletions and amplifications; to focal gene deletions or amplifications, such as *CDKN2A* deletion and *MYB* duplication [7], [8]; and to in-frame fusion genes encoding chimeric proteins with oncogenic properties such as the constitutively active *NUP214*-*ABL1* fusion kinase [9]. In addition, point mutations and small insertions/deletions (INDELs) have also been described leading to oncogenic events, such as mutations activating *NOTCH1* that occur in more than 60% of T-ALL cases [10], or mutations in cytokine receptors and tyrosine kinases such as *IL7R* and *JAK3* [11]–[17]. The latter may lead to new opportunities for molecularly tailored therapies with kinase inhibitors [12], [16], [18], [19].

With the advent of next generation sequencing (NGS) technologies, our sequencing capacity has significantly improved in the past five years. It is now possible to apply targeted re-sequencing, exome sequencing (Exome-seq), whole genome sequencing (WGS), whole transcriptome sequencing (RNA-seq) or a combination of these, to investigate individual genomes, especially those related to disease [20]. Also for T-ALL, these NGS approaches have recently proven their value in the discovery of novel driver genes [13], [14], [17], [21]. We previously identified a spectrum of new oncogenic driver genes using Exome-seq on 67 T-ALLs, and described clear differences between pediatric and adult patients [17]. In particular, we identified *CNOT3* as a tumor suppressor mutated in 8% of adult T-ALL cases and mutations affecting the ribosomal proteins *RPL5* and *RPL10* in 10% of pediatric T-ALLs [17]. Similarly, whole genome sequencing of early T-cell precursor ALL cases led to the identification of mutations in several new oncogenes and tumor suppressor genes affecting cytokine signaling, T-cell development and histone-modifying genes [2], [13]. However, the potential of RNA-seq for the discovery of driver genes in T-ALL remains unexplored.

In the present study, we applied paired-end RNA-seq on 49 T-ALL samples (31 patients, 18 cell lines) to gain insights in the transcriptome landscape of T-ALL. First, we show that identification of somatic single nucleotide variants (SNV) and recurrently mutated driver genes is feasible on RNA-seq data, even without matched normal samples (e.g., germlines or remission DNA). We identify *STAT5B, H3F3A*, and *PTK2B* as candidate cancer genes in T-ALL. This becomes possible when (1) optimal read mapping and SNV calling procedures are applied; and (2) functional annotation, gene expression, or additional sequencing data from other cohorts is used to prioritize the true driver genes. Next, we optimized gene expression measurements using multiple normalization strategies, and showed that classical gene expression studies (e.g., clustering) are feasible on normalized RNA-seq data. We also detected new fusion genes (*SSBP2*-*FER* and *TPM3*-*JAK2*) and used gene expression data to determine the consequence of observed chromosomal rearrangements on the over-expression of key driver genes. Finally, we searched for significant alternative transcript events (ATE) but besides one coherent exon-skipping event in *SUZ12*, we found relatively few candidate ATEs in T-ALL. In conclusion, through a combination of the analysis of gene expression levels, fusion transcripts, SNVs, and INDELs, we could identify known and new driver alterations in T-ALLs and novel potential targets for therapy.

## Results

### Correct SNV and INDEL calling on RNA-seq data depends on accurate read mapping

**Figure 1:**
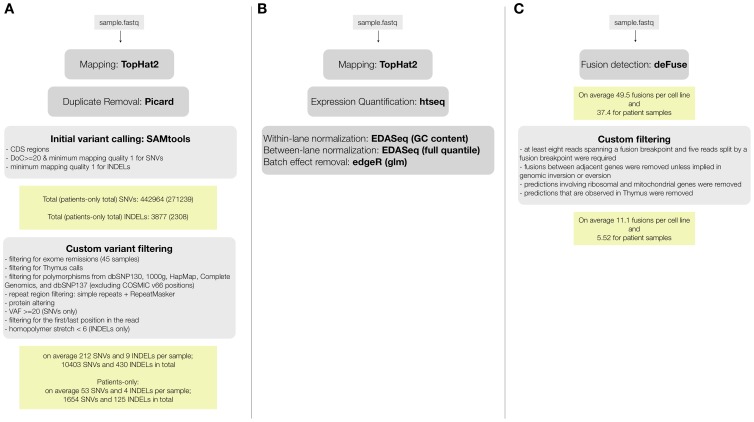
RNA-seq data analysis pipelines for (A) variant calling and filtering to detect point mutations, (B) fusion detection and annotation, (C) gene expression analysis.

We performed paired-end RNA-seq on 31 T-ALL patients, 18 T-ALL cell lines, and 1 normal thymus sample. We obtained on average ∼110 million reads per sample, leading to an average coverage of ∼88× (**Table S1.A**). To assess the quality of detecting SNVs from the RNA-seq data, we compared the RNA-seq to Exome-seq data. For 16/18 of the cell lines and for 20/31 patient samples we had exome data available (previously generated [17] or obtained for this study, **Table S2**). For the exome data analysis, we followed the pipeline of mapping, SNV and somatic mutation detection that we validated previously [17] (using BWA, GATK, SomaticSniper, and Variant Effect Predictor (VEP)) [22]–[25]. For the RNA-seq data we used TopHat2 [26] for mapping, SAMTools [27] for SNV detection, and VEP [25] for variant annotation (**Figure 1.A**).

**Figure 2:**
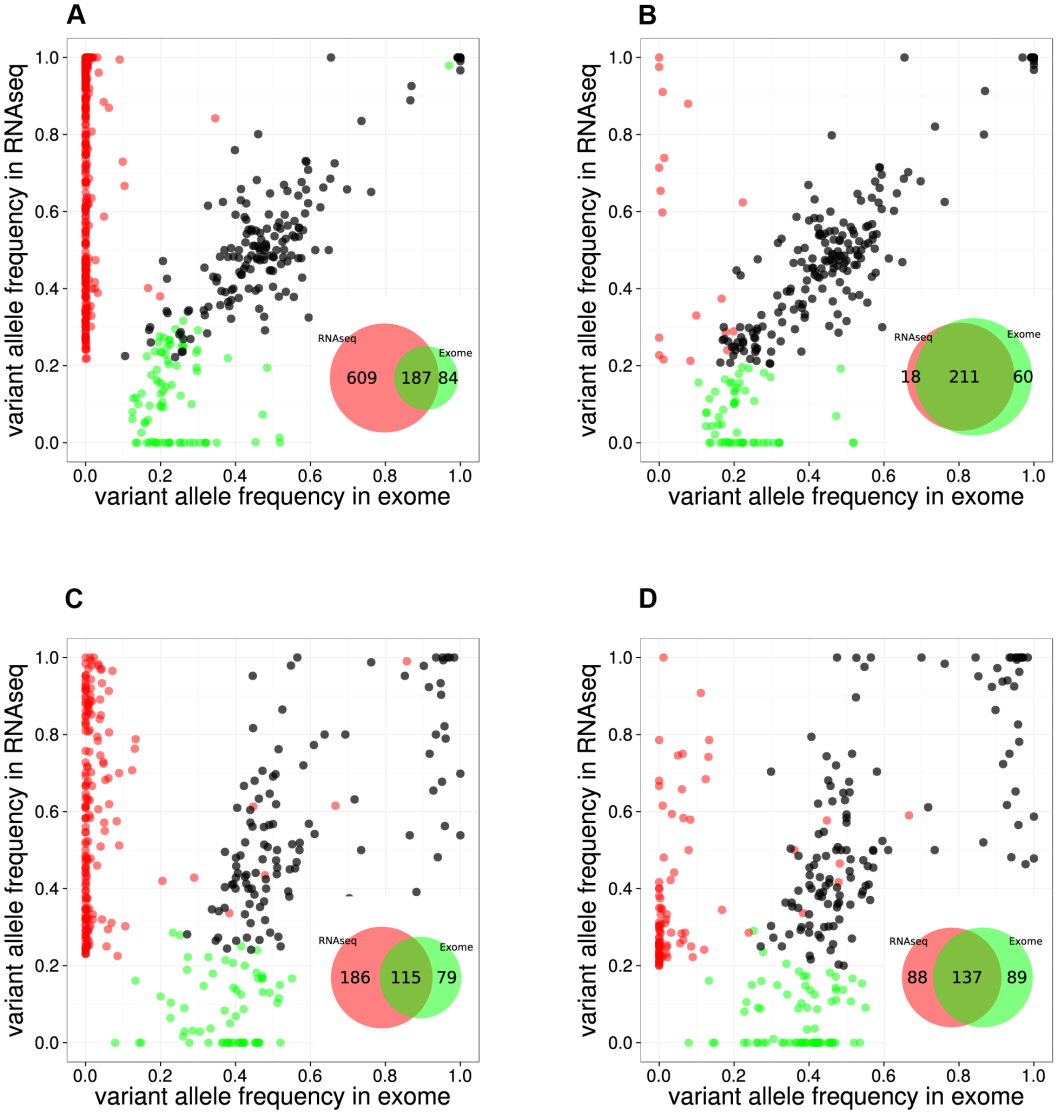
Comparison between RNA-seq and exome-seq. Variant Allele Frequency plots for evaluating two RNA-seq mapping strategies for two example samples, namely the RPMI8402 cell line (**A**, **B**) and the TLE79 patient sample (**C**, **D**). On the left are the results of mapping with TopHat 1.3.3. (**A**,**C**), while on the right are the results of mapping with TopHat 2.0.5 with forced re-mapping of all reads to the genome. The SNVs that have at least 20 reads in exome-seq and RNA-seq are plotted. Red and green dots represent the SNVs that are detected only in RNA-seq and only in exome-seq, respectively, while black dots represent the SNVs that are called in both. Venn diagrams are produced from the points represented in the graphs.

By comparing positions that had a coverage of at least 20× in both RNA-seq and Exome-seq, combined with Sanger re-sequencing of a subset of positions, we found that the accuracy of SNV calling in RNA-seq strongly depends on the read mapping, corroborating earlier observations [28], [29] (**Figure S1**). We found that mapping RNA-seq reads to the genome (as used by TopHat version 1.3.3) is prone to errors when dealing with paralogous genes, as observed by the prediction of false positive SNVs in *KIF4A* and *GLUD1* due to erroneous mapping to *KIF4B* and *GLUD2* (both pseudogenes with no introns) (**Figure S1**). However, these errors were resolved by mapping to the transcriptome. In the case of the RPMI8402 cell line, 877 SNVs were found by mapping to the genome, while this number was reduced to 283 SNVs when mapping to the transcriptome. Mapping to the transcriptome did not only reduce the number of RNA-seq exclusive calls but also increased the overlap with the Exome-seq calls (**Figure 2** **, Figure S2**).

However, transcriptome mapping also has limitations as it relies on current gene and isoform annotation. We observed that a combination of transcriptome and genome mapping provides the best solution. It is important that all reads are mapped twice to the genome, independently of each other; once as entire read and once as split read. This has become possible in TopHat2 by setting the option “*read-realign-edit-dist*” to zero. Our analysis reveals that this mapping approach results in the best overlap of SNVs compared to exomes (**Figure 2** **, Figure S3**). This mapping strategy not only improves the alignment accuracy by preventing misalignment to pseudogenes, but also leads to identification of the most likely isoform structure of a gene by mapping the reads independently both to the transcriptome and to the genome and then selecting the best possible alignment.

Using the optimized mapping and filtering strategy we identified 436,974 SNVs across 49 samples. By using samples for which both the exome and the transcriptome were sequenced several aspects of SNV detection in RNA-seq data can be evaluated, such as sensitivity, specificity, and allelic imbalance. Regarding sensitivity, we found that on average, 32% of the SNVs that are called in Exome-seq were also called by the RNA-seq (**Table S3**). Similar ratios were observed when comparing validated somatic SNVs from Exome-seq/WGS to RNA-seq SNVs: 36% in a triple negative breast cancer study [30], and 41% in a lymphoma study [31]. We observed that the sensitivity varies considerably between samples, and strongly correlates with the average depth of coverage of the sample (**Figure S4**). Regarding specificity, we found that the remaining RNA-seq-only and Exome-seq-only SNVs (for positions where both have at least 20× coverage) are found mainly with a low variant allele frequency (VAF) and are therefore likely due to arbitrary VAF and coverage thresholds. For example, on the RPMI8402 and TLE79 samples, many RNA-seq-only SNVs (9/18 and 61/88 respectively) have a VAF below 40%. Regarding allelic imbalance, we found that of all heterozygous Exome SNVs with more than 20× coverage, the majority (2,914/4,043 or 72%) were also heterozygous SNVs in RNA-seq. Of the remaining SNVs, many (988/4,043) are homozygous reference in the RNA-seq (i.e., not detected). A small fraction we can almost certainly attribute to allelic imbalance, namely the 141/4,043 SNVs (3.5%) that are homozygous variant in the RNA-seq, indicating that for those only the variant allele is expressed (or the gene is only expressed in cancer cells that harbor the variant).

**Figure 3:**
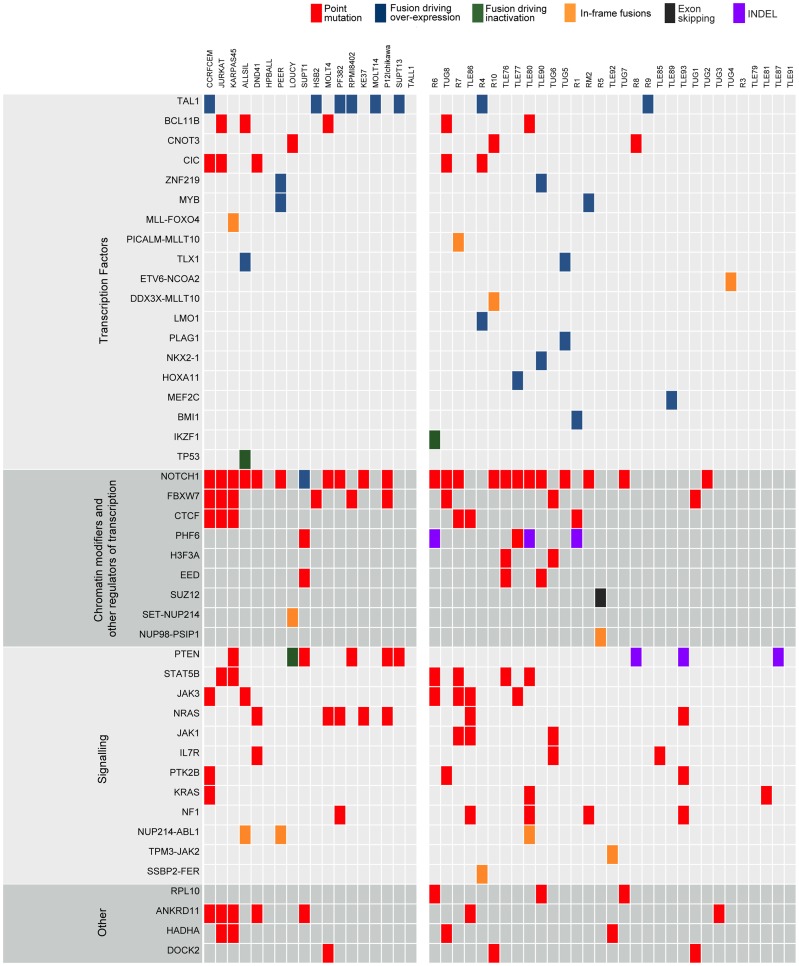
Point mutations and gene fusions organized into functional categories. Protein altering mutations and INDELs, alternative splicing events and validated fusions are shown. Red boxes indicate protein-altering mutations (i.e. nonsense, missense and splice site mutations); purple boxes indicate frame-shift INDELs whereas blue, green and orange boxes represent fusion events resulting in over-expression of the partner gene, inactivation of the partner gene or generation of a chimeric protein, respectively, and finally black boxes indicating alternative splicing events.

Next we asked whether small insertions and deletions (INDELs) can be detected from RNA-seq data. As with the SNVs, we used the Exome-seq data for assessing the quality of our INDEL detection strategy. On average, 47.5% of the INDELs that were detected by RNA-seq were also found in the Exome-seq (unfiltered) INDEL calls. However, only 4% of the Exome-seq INDELs (for which the region containing INDEL is covered by at least 3 reads in RNAseq data) were found back in the RNA-seq calls (**Table S3**). To investigate this sensitivity issue, we evaluated ten validated INDELs that we previously identified with Exome-Seq [17](**Table S4**). Three of the ten INDELs were also identified in the RNA-seq data using the default SAMTools parameters (see Materials and Methods). Of the seven missed INDELS, two are found in a gene that is not expressed; another two are clearly present in the RNA-seq data when inspected manually with IGV, but did not reach the default threshold (see Materials and Methods); and the last three are effectively discordant between RNA-seq and Exome-seq, as they show only reads with reference sequence (**Figure S5**). Re-mapping of the reads with BWA [22] on the transcriptome followed by BLAT [32] on the genome improved the INDEL identification, now revealing the *KDM6A* INDEL in TLE87 and *PTEN* INDEL in TLE92, which were previously missed (**Figure S6.A–B**). It is notable that the combination of TopHat2 (to transcriptome only) and BLAT does not correctly detect these two INDELS (**Figure S6.C–D**). We conclude that INDEL detection on RNA-seq data is feasible, yet technically challenging and that the fraction of INDELs compared to SNVs is moderate (see also the next Section and Figure 3).

### Leveraging diagnosis-only RNA-seq data with the T-ALL body of knowledge to identify mutated cancer genes

Our next aim was to select candidate driver genes using the collected SNVs and INDELS. To remove germline variants we initially removed all SNPs present in dbSNP [33], 1000genomes [34], the Complete Genomics genomes [35], and those detected in our own exome data from normal samples (39 from our earlier work [17] and 6 from this study). We, however, retained those variants also present in the COSMIC [36] database, since SNP databases are known to contain also some disease-specific SNVs. Some examples of SNVs that are likely driver mutations, but that are also present in polymorphism databases are: *JAK3* A572V in R7, and *FBXW7* R425C in TUG1. With this filtering, we obtained a final list of 10,403 protein-altering SNVs and 430 protein-altering INDELs, with a median of 63 SNVs and 4 INDELS per sample (**Table S1.B**). Cell lines harbored significantly more mutations than patient samples (Mann-Whitney test p-value = 1.095E-05), as previously also observed by Exome-seq [17].

As a first approach to identify candidate T-ALL driver genes, we selected all genes that contained a protein-altering mutation in at least two of the 31 patient samples (for recurrence we did not take cell lines into account). This process resulted in the selection of 213 genes (**Table S5**). We found that this list is strongly enriched for genes related to T-ALL and to cancer in general, with “precursor T-cell lymphoblastic leukemia-lymphoma” as the most highly enriched function (p-value = 1.35E-11 by Ingenuity Pathway Analysis) (**Table S6**). The list of 213 candidates contained many known T-ALL driver genes (**Figure 3**), such as *NOTCH1, BCL11B, FBXW7, IL7R, JAK1* and *JAK3*; and it also contained the drivers *CNOT3* and *RPL10*, recently identified in our exome re-sequencing study [17]; and *CTCF*, which was recently reported to be recurrently mutated in ETP-ALL [13]. In addition, the candidate list contained two established cancer driver genes involved in other cancer types, but not yet reported to be mutated in T-ALL, namely *H3F3A* and *CIC*. These genes were reported recently by Vogelstein [37] to be true cancer drivers. We identified two patient samples (TLE76 and TUG6) with *H3F3A* mutations both on the K28 residue that is a mutational hotspot in glioblastoma [38]. This mutation was confirmed somatic in the TUG6 sample. Sequencing of this hotspot in additional T-ALL samples indicated a low frequency of *H3F3A* K28 mutation in T-ALL (detected in 3 of 102 cases).

Next we asked if we could identify additional genes in the candidate list that could be linked to T-ALL. We wanted to utilize the genes that are known to be involved in T-ALL as a guide for identifying additional candidates. To this end we used our gene prioritization approach ENDEAVOUR [39], which scores candidate genes based on a set of training genes. It builds a profile based on the training genes (integrating information on protein-protein interactions, genetic interactions, gene expression, text-mining, sequence homology, Gene Ontology, and protein domains) and then prioritizes the candidate genes for their similarity to the derived profile. As training set we used all known drivers, and as test set we used all the 213 candidates with at least two patient mutations (excluding the genes that are in the training set). We reasoned that this would reveal the genes with strong similarity to the known drivers and such genes would be good candidate drivers. We found 45 significantly ranked genes with two interesting genes at the top of the ranking, namely *PTK2B* and *STAT5B* that are involved in JAK/STAT signaling (**Table S7**). Furthermore, the list contained genes for which we had identified single T-ALL cases with a somatic mutation in our previous exome study: *ANKRD11*, *CTCF*, *DOCK2*, *H3F3A*, and *HADHA*. We did not select these genes before in our Exome-seq cohort [17] because they were only mutated in one of the 39 samples we analyzed. Now, with the RNA-seq cohort, we thus found additional samples with mutations in these genes.

### Optimized gene expression measurements and batch effect removal from RNA-seq data identify co-expression modules and T-ALL subtypes

T-ALL is characterized by the overexpression of transcription factors (TFs), such as *TLX1, TLX3, TAL1*, and the *HOXA* family members [6]. Therefore, identifying and analyzing expression perturbations in a T-ALL cohort is highly relevant. To obtain accurate gene expression levels from the mapped RNA-seq reads, we followed the procedure outlined in **Figure 1.B**, including read aggregation, GC-normalization, length normalization, and between-sample normalization (see Materials and Methods). In addition, we removed a batch effect that was clearly present in the data set using a Generalized Linear Model (GLM, see Materials and Methods) (**Figure S7**). It is notable that transcript-based expression analysis conducted with *cufflinks* revealed the same batch effect linked to the origin of the sample, thereby confirming a technical bias in the data set (**Figure S7.B**, see Materials and Methods).

**Figure 4:**
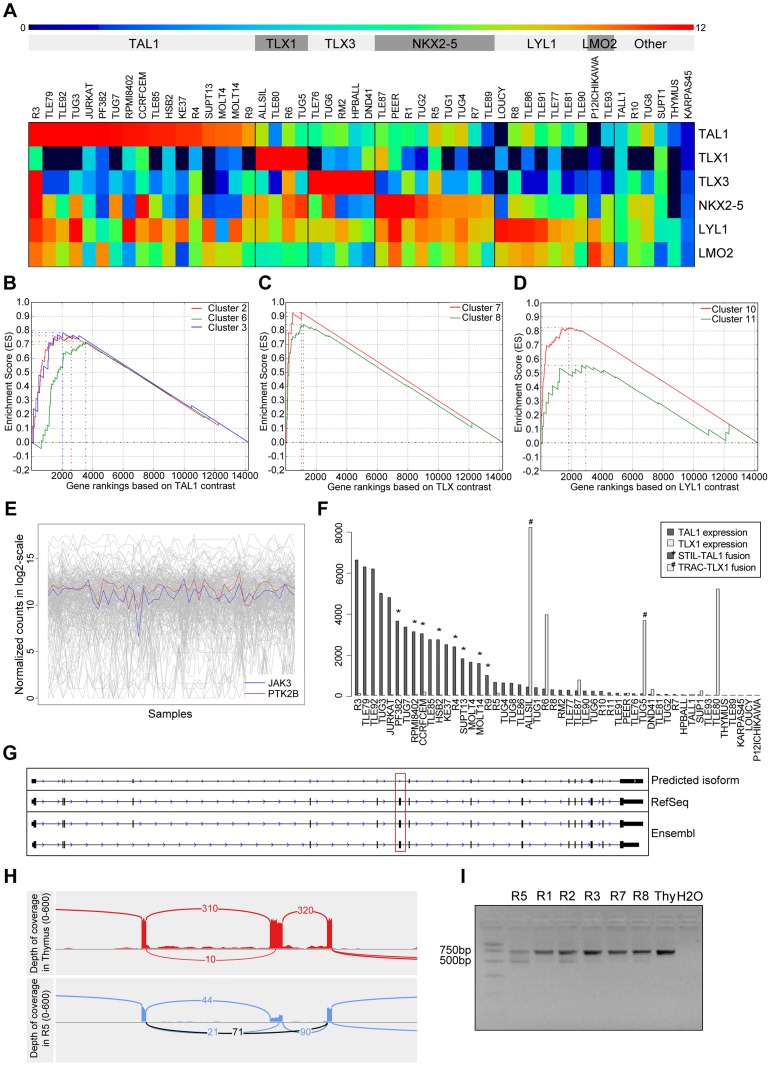
Validation and discovery using gene expression data, and *SUZ12* ATE. (**A**) Classification of the samples using the TFs that are known to be overexpressed in T-ALL. Using the expression patterns of *TAL1, TLX1, TLX3, NKX2-5, LYL1* and *LMO2* we could discriminate the samples in to six distinct clusters. The heatmap is plotted with the normalized log2(count) values. Gene set enrichment analysis curves are displayed for (**B**) enrichment of TAL1 associated clusters 2, 6 and 3 in *TAL1* based ranking, (**C**) enrichment of TLX associated clusters 7 and 8 in TLX based ranking, and (**D**) enrichment of *LYL1* associated clusters 10 and 11 in *LYL1* based ranking of the genes. (**E**) Expression of *JAK3* and *PTK2B* across samples is significantly correlated (with PTM p-value = 1E-05). (**F**) Normalized expression values of *TAL1* and *TLX1* with translocations affecting these genes indicated. The samples with a translocation have elevated expression of the affected gene, showing the driver potential of the fusion event. There are additional samples with high expression of *TLX1* and *TAL1* without the indicated fusions, pointing to other mechanisms of activating these genes. (**G**) Predicted *SUZ12* transcript aligned with the known *SUZ12* isoforms. Dotted red box indicates the location of the exon-skipping event. (**H**) The sashimi plot shows the junction (in black) supporting the exon-skipping event in patient sample R5 with respect to Thymus. (**I**) Agarose gel electrophoresis of the RT-PCR products for validation of *SUZ12* exon skipping event. The two isoforms are clearly detected in R5 and to a minor extent in the other T-ALL samples while Thymus shows only the canonical transcript.

We next looked at the expression values of *TLX1*, *TLX3*, *TAL1*, and other important TFs in T-ALL. Clustering of *TLX1, TLX3*, and *TAL1* expressing samples confirmed that the correct samples (based on karyotyping and molecular analysis) showed over-expression of the respective TF (**Figure 4.A**). Indeed, 8 samples that harbored a *STIL-TAL1* rearrangement showed high *TAL1* expression (**Figure 4.D**). Note that also other samples with high *TAL1* expression were detected. This fits with a previously reported observation of *TAL1* over-expression in the absence of a translocation in T-ALL [6], [40].

To assess the accuracy of our expression values obtained after normalization, batch effect removal and clustering, we tested whether previously published gene signatures associated with *TAL1*, *TLX* (*TLX1* and *TLX3*) and *LYL1* can be detected also in our data set [41]. We used 13 gene signatures obtained by Soulier *et al* using a microarray study on 92 primary T-ALL samples [41]. Gene set enrichment analysis shows that our *TAL1* expressing cases are significantly associated with *TAL1* signatures, whereas our *TLX* over-expressing cases are associated with the *TLX* signature [7], [8] and the *LYL1* cases with the *LYL1* signature [10], [11]. This analysis confirms that the obtained expression data represent meaningful values and sample clustering produces gene lists that are biologically meaningful (**Figure 4.B**).

We next used the gene expression information as a guide to assist in the detection of relevant mutations. We found that the expression profile of *PTK2B*, a candidate driver identified above by ENDEAVOUR, significantly correlated with the *JAK3* expression profile (PTM, with p-value threshold at 1E-05, see Materials and Methods) (**Figure 4.C**). Indeed, *PTK2B* was previously implicated in *IL-2* mediated signaling and JAK/STAT signaling, and was shown to physically interact with *JAK3* [42]. These data warrant further investigation of *PTK2B* as an important tyrosine kinase in T-ALL case with activated JAK/STAT signaling.

### T-ALL presents robust transcript isoform usage

To our knowledge, only very few cancer specific alternative transcript events (ATE) have been described for any cancer type [43]–[45], and no ATE is reported for T-ALL. In contrast to SNVs, INDELS, copy number variations, and fusions, which are all curated and present in large numbers in public cancer mutation databases (e.g., COSMIC [36], CENSUS [46]), we could not find driver ATEs in those databases (although splice sites represent an important class of cancer mutations). If ATEs represent an important, yet underestimated, type of somatic variation in cancer, we would expect at least some of the known cancer driver genes to present a significant ATE. We thus asked whether novel variations could be found in these genes in the form of ATEs. To this end, we applied *cufflinks* and *cuffdiff* (see Materials and Methods) and found significant ATEs in 12 of the 47 known driver genes (*BCL11B, FLT3, IL7R, LCK, MYB, NKX2-1, SFTA3, RPL10, RUNX1, SETD2, SUZ12*, and *TAL1*) (**Table S8**). However, when we manually inspected these events in IGV, we found only two interesting cases. One case represents an unambiguous skipping of exon 7 in *SUZ12*, occurring in several patient samples, but most significant (cuffdiff p-value = 5.10E-05) in the R5 patient sample, and absent in the Thymus (**Figure 4.E**), and a potential, but less clear, skipping of exon 8 in *LCK* in three samples (**Figure S8**). Exon 7 of *SUZ12* is a canonical exon (present in all known isoforms) according to RefSeq, Ensembl, and UCSC annotation. The ATE we observe is a heterozygous event with the wild-type junction supported by 90 reads and the novel junction supported by 71 reads. RT-PCR clearly confirmed the exon-skipping event in R5 and to a minor extent in other samples, while being absent in the thymus (**Figure 4.F**). The functional consequences of these splice variants remain to be determined, but the fact that these variants are both in-frame suggests that these proteins could be functional protein isoforms (**Figure S8 and S9**). Overall, relatively few significant ATEs are detected, and no obvious ATEs are found with consequences on the protein structure, therefore T-ALL presents robust isoform usage at the current resolution of sequencing and analysis.

### Detection and validation of known and novel fusion transcripts

Most of the T-ALL cases harbor chromosomal rearrangements that lead to the generation of fusion genes or ectopic expression of genes due to juxtaposition to strong promoters or regulatory sequences. Chromosomal translocations involving the TCR genes are largely underestimated by karyotyping and the TCR partner genes remained unidentified in several cases [4], [47]. On the other hand, a multitude of mechanisms other than translocations could cause ectopic expression of oncogenes [48]. To detect fusion transcripts, we used the defuse algorithm on our entire dataset [49]. Briefly, this method identifies candidate gene fusions by discordant alignments produced by spanning reads (each read in the read pair aligns to a different gene) and by split reads (reads that harbor a fusion boundary). The total number of predicted fusions initially was 1,160 and 1,265 in patient and cell line samples, respectively. Also in normal thymus RNA, 60 fusion transcripts were detected. Next, we implemented additional filters, considering only predictions supported by 8 or more spanning reads and 5 or more split reads. Furthermore, we removed fusions involving ribosomal genes, mitochondrial genes and fusions between adjacent genes, as these could be caused by read-through or trans-splicing [50], [51] (**Figure 1.C**).

After applying these filters, we obtained an average of 5.5 fusion events per patient sample and 11.1 per cell line (**Table S1.C**). In total, 397 candidate genes are involved as potential partner in a gene fusion (**Table S9**). Details on the fusion breakpoints and validation of the novel candidate fusion transcripts are reported in Tables S9 and S12 (see also Materials and Methods: RT-PCR and Sanger Sequencing).

First, to determine the relevance of these predicted fusion transcripts we looked at functional enrichment of these genes. 278 of 397 genes correspond to functionally annotated protein-coding genes according to DAVID functional enrichment [52], [53]. Furthermore, this set is strongly enriched for cancer-related genes, and more specifically for genes involved in Acute Myeloid Leukemia (p-value = 4.48E-10) and T-ALL (p-value = 4.47E-05), including *TP53, STAT5B, NOTCH1, IL7R, IKZF1, CDKN2A, MLLT10, ETV6*, and *ABL1*.

Second, we specifically analyzed the 27 in-frame fusions, predicted to encode chimeric proteins (**Table S10**). This list contained known oncogenic fusion genes, including *NUP214-ABL1* (n = 2), *MLL-FOXO4* (n = 1), *PICALM-MLLT10* (n = 1), *ETV6-NCOA2* (n = 1) and *SET-NUP214* (n = 1). In addition, we identified 3 novel chimeric transcripts in T-ALL, namely *NUP98-PSIP1* (n = 1), *TPM3-JAK2* (n = 1) and *SSBP2-FER* (n = 1) and a novel *DDX3X-MLLT10* fusion transcript (n = 1) recently described in a pediatric T-ALL patient [54]. Conventional cytogenetic analysis confirmed the presence of a t(X;10) in the case with the *DDX3X-MLLT10* fusion, whereas it failed to detect the chromosomal rearrangements for the *TPM3-JAK2, NUP98-PSIP1* and *SSBP2-FER* fusions, demonstrating the power of RNA-seq to identify cryptic fusion genes and to provide genetic information even in patients with uninformative cytogenetics. Reassuringly, RT-PCR and Sanger sequencing confirmed the presence of these fusion transcripts (**Table S12**).

**Figure 5:**
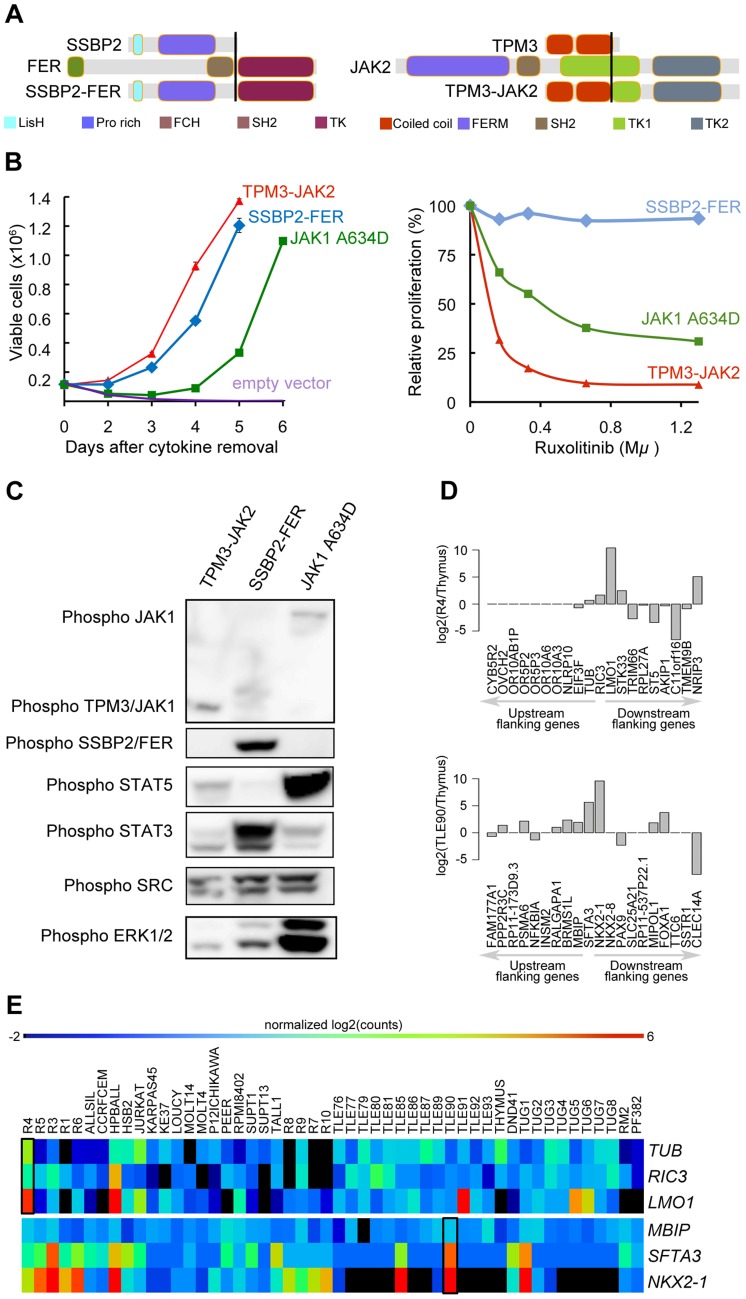
*SSBP2-FER* and *TPM3-JAK2* fusions transform lymphoid cells and show constitutive activity. (**A**) Schematic representations of the predicted *SSBP2-FER* and *TPM3-JAK2* fusion joining the dimerization units of SSBP2 (LisH domain) or TPM3 (coiled-coil domains) to the TK domain of FER or JAK2, respectively. (**B**) Proliferation curve of mouse Ba/F3 cells in the absence of the cytokine interleukin 3 (*IL3*) (upper graph) and in the presence of ruxolitinib (lower graph). In the absence of IL3, cells expressing empty vector died whereas cells expressing the *SSBP2-FER* or *TPM3-JAK2* fusion protein were transformed and could proliferate. Ba/F3 cells expressing the oncogenic *JAK1* A634D mutant were used as positive control for transformation [18]. The graph shows mean +/− st. dev. The lower graph illustrates the effects of the *JAK* kinase inhibitor ruxolitinib on Ba/F3 cell proliferation after 24 hours of treatment. The graph represents mean +/− st. dev. of triplicate measurements. (**C**) Western blot analysis of Ba/F3 cells transformed by the indicated kinases. The 2 upper panels show phosphorylation of the *JAK* and *FER* kinases, the panels below illustrate phosphorylation of downstream targets *STAT5*, *STAT3*, *SRC* and *ERK1/2*. (**D**) TCR gene fusions result in overexpression of a flanking gene in *RIC3-TRBC2* and *SFTA3-TRDC* fusions. The barplot is drawn for relative (to Thymus) expression values for the upstream and downstream flanking genes around *RIC3* and *SFTA3* for R4 and TLE90 samples, respectively. In both cases, the nearest downstream neighbor shows increased expression. (**E**) The heatmap illustrates the expression patterns of *RIC3* and *SFTA3*, together with their immediately upstream and downstream flanking genes in the genome, showing strong over-expression (red) of *LMO1* near the *RIC3* fusion, and of *NKX2-1* near the *SFTA3* fusion.

The *TPM3-JAK2* and *SSBP2-FER* fusions encode typical tyrosine-kinase fusions that join the tyrosine-kinase domain of *JAK2* or *FER* to the dimerization units of *TPM3* or *SSBP2*, respectively (**Figure 5.A**). To assess whether the *TPM3-JAK2* and *SSBP2-FER* fusions encode oncogenic proteins, we tested their transforming properties in the *IL-3*–dependent Ba/F3 cell line [55]. Both *TPM3-JAK2* and *SSBP2-FER* transformed Ba/F3 cells to *IL-3*–independent growth, with even faster kinetics than the *JAK1* A634D mutant, which is a known transforming kinase [18] (**Figure 5.B**). Western blot analysis confirmed the constitutive auto-phosphorylation of the *JAK2* and *FER* fusion proteins, as well as the downstream STAT proteins (**Figure 5.C**). Ba/F3 cells transformed by the *TPM3-JAK2* fusion were sensitive to a JAK kinase inhibitor, documenting the potential application of *JAK2* kinase inhibitors for the treatment of T-ALL cases with *JAK2* fusion genes. No specific *FER* inhibitors were available to test their activity. Both *TPM3-JAK2* and *SSBP2-FER* fusion were screened in 50 additional T-ALL samples, but no additional case with these fusions was found.

Third, we also analyzed the identified fusions that did not seem to encode chimeric proteins (out-of-frame fusions), and which were the majority of fusions detected in T-ALL. These fusion events can be used as surrogate markers for the identification of chromosomal rearrangements, providing accurate information on the precise chromosomal breakpoints. In combination with the gene expression data obtained by RNA-seq, these data can identify genes that are located close to such potential breakpoints and for which the expression is significantly up- or down-regulated. As expected, we identified the *STIL-TAL1* fusion in several T-ALL cases (n = 8). We also identified and validated 6 fusion events involving TCR genes. In 4 of these cases, the TCR gene was found to be fused to the potential oncogene (*NOTCH1*, *IL7R*, *PLAG1*, and *TLX1*). In the two other cases (R4, TLE90), the TCR gene was fused to *RIC3* or *SFTA3*, resulting in the ectopic expression of *LMO1* and *NKX2-1*, respectively, as indicated by RNA-seq gene expression data (**Figure 5.D and E**). Similarly, we could better characterize the t(10;14) in ALL-SIL cell line that expresses *TLX1* at high level.

In addition to the TCR gene rearrangements, also other fusions were associated with overexpression. We detected out-of-frame fusion transcripts that joined exon 4 of *CDK6* to exon 2 of *HOXA11-AS* and exon 5 of *CDK6* to sequences downstream of *EVX1*. In the same patient we also detected a fusion joining *DPY19L1* on chromosome 7p14 to *HOXA11* on chromosome 7p15. The gene expression analysis documented high expression of genes of the *HOXA* cluster (i.e. *HOXA9, -A5, -A13, -A10, -A11*). Moreover, other fusions identified in this study, such as *CLINT1-MEF2C*, *HNRP-ZNF219* (n = 2), *ZEB1-BMI1* and *AHI1-MYB* (n = 2) were also associated with transcriptional activation of *MEF2C, ZNF219, BMI1* and *MYB* as confirmed by the expression data (**Table S9** and **S12**, and **Figure S10**). Increased *MYB* expression in T-ALL was previously observed as a consequence of *MYB* duplication (including in the BE-13 cell line), which may also explain the detected *AHI1-MYB* fusion [8], [56].

Finally, we also found out-of-frame fusion transcripts leading to the potential inactivation of tumor suppressor genes, such as *TP53-TBC1D3F* (ALLSIL cell line), *PTEN-RNLS* (LOUCY cell line), *IKZF1-ABCA13* and *CDKN2A-miR31HG* (R6 case), indicating a third class of fusion events (**Figure S10**). FISH analysis performed in the R6 case confirmed the p15/p16 deletion. As the genes are in close proximity, the *IKZF1-ABCA13* was presumably generated by deletion although no material was available to confirm this hypothesis.

## Discussion

The landscape of genomic variation underlying T-ALL has recently been investigated by sequencing candidate genes [14], [21], whole exomes [17] and whole genomes [13]. The results of these studies, combined with a large body of gene-by-gene evidence collected over the last decade, provide a growing comprehension of the T-ALL genome. The T-ALL genome is mainly characterized by the over-expression of TF, such as *TLX1/3* and *TAL1*, in combination with gain-of-function *NOTCH1* mutations, and with additional mutations in chromatin modifiers, cellular signaling factors such as those involved in the JAK-STAT signaling pathway [57], tumor suppressor genes (*TP53, PTEN, WT1*), or in other genes such as ribosomal genes [17]. Since the majority of observed mutations are point mutations and gene fusions (much more than copy number variations [13]) we reasoned that RNA-seq would be effective to identify many of these mutations, certainly those associated with (over-)expressed oncogenes. Indeed, exome sequencing allows identifying point mutations but not gene fusions; and low coverage whole-genome sequencing allows identifying structural variation (gene fusions) but not point mutations. In this study we present RNA-seq analyses on a heterogeneous group of 31 T-ALL samples and 18 T-ALL cell-lines and demonstrate that RNA-Seq is indeed a very powerful approach to detect gene mutations and fusions as well as expression perturbations.

Our first challenge with regards to the accurate identification of point mutations was finding the optimal analysis pipeline – from read mapping to SNV calling and filtering – to avoid too many false positive SNVs. By exploiting whole-exome sequencing data for a subset of our samples we obtained a recovery ratio of 32% when compared to the exome derived SNVs; a ratio that is comparable with previous RNA-seq studies [30], [31]. However, this concordance could only be achieved by using the optimal read mapping methods and parameters: (1) use of a recent version of TopHat2 (v. 2.0.5. or higher) and (2) forcing this aligner to map all reads twice to the genome (once directly and once using split reads) and once to the transcriptome. Indeed, the computational task of sequence read mapping is more challenging for RNA-seq data because a large fraction of the obtained reads need to be split to allow reads that overlap exon-exon boundaries in the cDNA to be mapped to the genome. In this way, RNA-seq is more prone to the identification of false SNVs due to the erroneous mapping of reads, for example to highly similar non-spliced pseudogenes. For example, in the RPMI8402 cell line, 603 RNA-seq exclusive SNVs were found with the genome mapping strategy, while only 35 when using combined mapping strategy.

Among the previously published large scale RNA-seq cancer studies, only a handful performed variant calling on the RNA-seq data [30], [31], [58], [59]. A combined mapping strategy was followed in all cases either by mapping the reads to a customized genome reference file (by the addition of exon junction segments) or mapping the reads twice (once to the genome and once to the transcriptome). Variant calling pipelines also showed diversity: Morin *et al* and Shah *et al* used SNVMix [60] for variant calling, while Seo *et al* and Berger *et al* implemented filters based on alignment on the non-reference bases. To our knowledge there is no extensive benchmarking study evaluating aligners and variant callers for RNA-seq data, but a review paper by Quinn *et al* compared the performance of two variant callers (GATK [23] and SAMTools [27]) with the optional duplicate removal step (pre and post alignment), and concluded that post-alignment duplicate removal and variant calling with SAMTools achieved the best performance in terms of sensitivity and specificity [61]. We have also followed the same strategy in our study and we could achieve a comparable recovery ratio of 32% when compared to Exome-seq calls.

A second challenge in identifying point mutations was the prioritization of candidate driver mutations versus passenger mutations. Due to the lack of matched germline RNA for each patient as control, we used a large cohort of local normal exome datasets, in combination with the commonly used variants from dbSNP and 1000genomes, to distinguish SNPs from candidate somatic mutations. This strategy has been successfully used before on transcriptome sequencing studies [62]. Identifying candidate cancer genes by gene mutation frequency is a frequently used approach [13], [30], [58]. Remarkably, by simply selecting all genes having a candidate somatic mutation in at least two samples (213 genes in total), we already achieved a highly significant enrichment for T-ALL related genes, such as *NOTCH1*, *BCL11B*, *FBXW7*, *DNM2*, *JAK3*, *JAK1*, and *IL7R*. Among the remaining candidates we searched for additional evidence and we propose seven additional candidate drivers because they are either “functionally similar” to the previously known drivers, or because they were mutated somatically at least once in another T-ALL cohort [17], or both. Six of these genes, namely *CIC*, *H3F3A, PTK2B, STAT5B, ANKRD1* and *HADHA* have already been implicated in other cancers [63]–[70] while *DOCK2* has no association with cancer yet.

We found a remarkable clustering of molecular functions among the identified T-ALL driver genes, with enrichment for functions related to the regulation of gene expression. TFs and their co-factors play a central role in transcriptional regulation and these proteins are often mutated in T-ALL. Also, many of these play important roles in the normal T-cell developmental gene regulatory network [71], such as *NOTCH1, TLX1, TLX3, TAL1, BCL11B, CTCF, FOXO4, MYB*, and others. Upstream of these activated TFs, multiple kinases and other signaling factors control their activity, and these regulators are also often mutated in T-ALL (for example, *JAK1, JAK3*, and *IL7R*). Finally, chromatin modifiers and methylation factors are recurrently mutated and these can have both generally pervasive but also specific effects on the expression of oncogenes, such as *MYC* [72]. When multiple driver mutations are serially acquired, their combined effect will result in oncogenic expression profiles, whereby genes supporting a growth advantage increase and genes negatively affecting growth advantage (e.g., apoptosis, senescence) decrease in expression. It will be an interesting future challenge to draw the connections between the observed DNA mutations, the oncogenic program, and the final gene expression changes that we and others observe in T-ALL samples. Finally, it is likely that non-coding mutations, such as those in promoters, enhancers, microRNAs, and lncRNAs, add to the cancer-related gene regulatory network changes underlying leukemogenesis.

As mentioned above, only mutations in genes that are actively transcribed are detected, and this likely adds to the specificity of driver gene detection. On the other hand, this could also present a limitation of RNA-seq, because loss-of-function mutations in tumor suppressor genes may lead to nonsense-mediated decay, and as consequence low sequence coverage to call mutations. Based on our data however, this is not the case because we could detect *PHF6* mutations in up to 4/31 patient cases (13%), where exome sequencing identified *PHF6* mutations in 9/67 cases (13%) [17] and Zhang *et al* identified *PHF6* mutations in 24/106 cases by means of whole genome sequencing and capillary sequencing [13].

Interestingly, the gene expression information used above (i.e., read coverage to identify point mutations) can be further exploited at the quantitative level, similar to gene expression studies performed with microarray technology over the last 15 years. As many leukemia driver genes are characterized by changes in gene expression, this level of information is invaluable, both in research and diagnostic settings. We investigated how accurate gene expression levels can be achieved and we found that multiple normalization steps are required, both within-sample (gene length and gene GC content) and across samples (library size), and that batch effects can be effectively removed using a previously published Generalized Linear Model (GLM) [73]. The gene expression levels of the known drivers (e.g., *TLX1/3, TAL1, NOTCH1*) are highly representative as driving events and as subtype identifiers. However, to discover driver genes *de novo*, using only gene expression values, is to our opinion not feasible (data not shown). Alternatively, we attempted to select candidate drivers based on the expression similarity (i.e., co-expression across the cohort) with known drivers. This led to the identification of *PTK2B*, whose expression strongly correlated with *JAK3* and which is known to be implicated in JAK-STAT signaling. The next level of gene expression analysis would preferably be a network-level analysis [74], but this requires a larger sample cohort.

Another kind of information that can be extracted from RNA-seq data, besides point mutations and gene expression changes, are alternative transcript events (ATE) and gene fusions [75]. We found only few significant ATEs but could confirm two exon-skipping events in the known T-ALL oncogenes *SUZ12* and *LCK*. More importantly, we identified (i) known and novel in-frame fusions encoding chimeric proteins, (ii) TCR gene arrangements resulting in over-expression of oncogenes, and (iii) fusions not involving TCR genes but also resulting in over-expression of oncogenic transcription factors. The most recurrent fusion event, observed in 8/31 samples, was the *STIL-TAL1* fusion resulting in the ectopic over-expression of the *TAL1* gene. We also identified novel gene fusions, including two in-frame fusions, *TPM3-JAK2* and *SSBP2-FER*, producing chimeric oncoproteins; and other fusions resulting in the ectopic expression of transcription factors such as *PLAG1, MEF2C, ZNF219*, and *BMI1*. The ectopic expression of these genes is associated with a fusion event and with changed expression, which can both be detected by RNA-seq, making this technology extremely powerful to accurately detect such oncogenic events. Each of these novel events appears to be rare in T-ALL, as we identified at most 2 cases of each fusion. However the evidence of transcriptional activation of the partner genes suggests that further studies are required to establish the recurrence of these lesions and their functional meaning. It is notable that the normal thymus sample also shows four fusion events. However, as these genes are located in close proximity to each other, they may represent unannotated isoforms in the human transcriptome. Despite RNA-seq has offered a deeper insight into the complexity of the transcriptome, several studies have highlighted that the catalogue of all expressed transcripts is still far from complete and it is increasing the number of novel splice junctions connecting novel exon, non-exon regions, or linking independent transcripts [76].

Today, high-quality catalogues of driver genes across cancer types are available, and this influences how and why cancer genomes need to be sequenced. For T-ALL, and for many common cancer types, the objectives of sequencing are shifting from the discovery of cancer genes, to a diagnostic setting in which a list of driver events are *a priori* known. Targeted re-sequencing provides an interesting route, although this poses technical challenges of amplification or capturing, and perhaps more importantly, is focused on a limited number of genes and on one particular mutation type, namely point mutations and small insertions/deletions. We have shown in this study that, with a list of interesting cancer drivers at hand, and with other datasets being available (e.g., rare variants from local exome studies, 1000 genomes, TCGA data, etc), RNA-sequencing of only the cancer sample provides a technically straightforward approach and delivers at once the point mutations, gene fusions and gene expression changes across the entire transcriptome. And as a corollary, the data analysis strategies provided here would be beneficial for any cancer type as long as a body of knowledge is available for selecting and prioritizing candidate events.

## Materials and Methods

### Patient samples and cell lines

Diagnostic total RNAs from 31 T-ALL patients (20 adults and 11 children) were collected at various institutions. All patients have given their informed consent and all samples were obtained according to the guidelines of the local ethical committees. This study was approved by the ethical committee of the University Hospital Leuven. Diagnosis of T-ALL was based on morphology, cytochemistry and immunophenotyping according to the World Health Organization and European Group for the Immunological Characterization of Leukemia criteria [77]. The clinical and hematologic features of the 31 patients at the diagnosis are summarized in Table S11 Total RNAs from 18 T-ALL cell lines (DSMZ, Braunschweig, Germany) were extracted using QIAGEN RNeasy Mini Kit. A pool of total RNAs from 5 normal human thymuses was purchased from Capital Biosciences.

All the RNA samples showed a high quality RNA Integrative Number (RIN>/ = 7) score on the Bioanalyzer (Agilent Technologies).

Fifty additional RNA samples were used for *TPM3-JAK2* and *SSBP2-FER* analysis.

Genomic DNA from of 71 adult T-ALL patients were used for *H3F3A K28* screening.

### RNA-seq

Next generation sequencing libraries were constructed from 500 ng of total RNA using the Truseq RNA sample prep kit (Illumina). RNA-seq libraries were subjected to 2×100 bp paired-end sequencing on a HiSeq2000 instrument (Illumina). Sequence reads were processed to identify gene fusion transcripts, single nucleotide variants (SNVs) and gene expression levels. For the read mapping, variant calling and transcriptome assembly, we used the infrastructure of the VSC - Flemish Supercomputer Center, funded by the Hercules foundation and the Flemish Government - department EWI.

### Fusion transcript discovery

Fusion transcript discovery was performed using defuse v.0.5.0 [49] with default parameters. The resulting list was filtered as described in [78]. Briefly, fusion transcripts with less than 8 spanning reads and less than 5 split reads were filtered out. In addition, we removed fusion events observed in adjacent genes and fusion events involving ribosomal genes (ribosomal genes were downloaded from Biomart on 24-05-2011 using GO:0005840) and the genes located on chrM. Fusion events were annotated using Pegasus (http://sourceforge.net/projects/pegasus-fus/).

### Gene expression analysis

For Gene Expression Profiling analysis, reads were mapped to the human reference genome (assembly GRCh37.68) using TopHat v.2.0.5 [26] with the following parameters: transcriptome-only. Read counts per gene were obtained with the HTSeq package (htseq-count) (http://www-huber.embl.de/users/anders/HTSeq). The aggregated read counts were normalized with EDASeq v1.4.0 [79] and generalized linear model was fitted with edgeR v3.0.4 [73] to remove batch effect originating from the sample collection center. The pathways, and upstream regulators were generated through the use of IPA (Ingenuity Systems, www.ingenuity.com). Expression neighbors were detected with Pavlidis Template Matching (PTM) analysis [80]. Transcript based gene expression values were obtained using cufflinks suite [81], [82]. Transcript assembly was performed with cufflinks v2.1.1 with –g option using assembly GRCh37.68.

Gene set enrichment analysis (GSEA) was performed for *TAL1*, *TLX* and *LYL1* clusters [83]. We have obtained whole genome rankings for *TAL1*, TLX (*TLX1* and *TLX3*), and *LYL1* simply by calculating the log fold changes between samples expressing the respective gene versus the remaining samples. The gene signatures from Soulier *et al* were obtained from Table S2 [41].

### Alternative transcript event discovery

Tumor patient samples and Thymus RNA-Seq samples were mapped to the Ensembl GRCh37.68 reference genome by Tophat2 [26]. Mapped reads were realigned, and transcript abundance were estimated using cufflinks v2.1.1 [81], [82]. Transcript assembly was reconstructed using the *cuffmerge* program of the *cufflinks* package from the realigned transfrags for each of patient RNA-seq samples, merged with the Thymus sample (control), followed by differential expression analysis performed using *cuffdiff* program. The significant events were extracted from the list of differentially expressed genes, isoforms, primary transcripts and coding sequence and assessed manually with IGV [84]. The mRNA sequences for novel *SUZ12* and *LCK* transcripts were extracted using *gffread* command of cufflinks, and these sequences were translated using the *translate* tool of the ExPASy Bioinformatics Resource Portal [85]. The longest ORF sequence was used to verify the domain architecture of the resulting proteins using SMART [86], [87].

### Prediction of single nucleotide variation

The sequence reads were mapped to the human reference genome (assembly GRCh37.68) using TopHat2 setting the option “read-realign-edit-dist” to zero [26]. Duplicate removal process was performed on the aligned reads using Picard v1.74 (http://picard.sourceforge.net). Then SAMTools package v0.1.19+ (pulled from the git repository on 29-07-2013) [27] was used for single nucleotide variant (SNV) and small insertion and deletion (INDEL) detection with minimum mapping quality threshold of 1 and minimum base quality threshold of 13 (-q 1 -Q 13) [27]. The variant calling was done on the coding regions of the genome only (extracted from the transcript definitions in the assembly GRCh37.68). The variant predictions that were supported exclusively by variants located in the beginning or the end of the read were filtered out. Then the SNVs were further filtered with depth of coverage threshold of 20 and minimum variant allele frequency threshold of 0.20. INDELs predictions were filtered with the SAMTools recommended parameters (varFilter -10 -20 -30 -40 -a4 -G90 -S30) and additionally INDELs located in homopolymer stretches longer than 5 bps were filtered. The high quality list of variants was filtered for common population variants using the calls from 1000 genomes, dbSNP, HapMap, and Complete Genomics. Note that, the list of common population variants was cleaned from oncogenic variants using COSMIC listed variants (v66) [36]. Moreover, the variants located in the repeat regions (simple repeat and RepeatMasker) were filtered out. Finally, the variants that are observed in the exomes of remission (i.e. healthy) samples (including the previously published 39 exome remissions [17] and the 6 additional exome remission sequenced) and the variants that are observed in Thymus were also filtered out. The final filtered list of variants was annotated with the Variant Effect Predictor version 2.7 [25] and the protein-altering mutations were selected. The following terms were used for selecting protein-altering SNVs: splice-donor-variant, splice-acceptor-variant, stop-gained, initiator-codon-variant, missense-variant, splice-region-variant. The same terms were used for filtering the INDELs with the addition of the following terms: inframe-insertion, inframe-deletion, frameshift-variant.

The list of candidate genes was created by intersecting the genes with recurrent mutations (SNVs and INDELs) in RNA-seq patient cohort with the somatic mutations in Exome-seq patient cohort [17]. The list of genes that have recurrent mutations in the RNA-seq patient cohort was filtered for mutations observed in chrM.

The list of T-ALL driver genes were curated using the Census database [46] and T-ALL literature and includes the following genes: *TLX1, TLX3, PHF6, MYC, BCL11B, HOXA1, SET, MLL, MLLT1, PICALM, MLLT10, WT1, MYB, LEF1, LMO2, LMO1, TAL1, NUP98, NOTCH1, FBXW7, CCND2, PTEN, PTPN2, NF1, FLT3, JAK1, NRAS, LCK, NUP214, ABL1, EZH2, SETD2, SUZ12, JAK3, MEF2C, NKX2-1, NKX2-2, CDKN2A, CDKN2B, RUNX1, KRAS, EED, ETV6, RPL10, DNM2, IL7R, CNOT3*.

### Exome-seq analysis

Somatic mutations from the exome pairs were obtained as described previously [17]. Briefly, the alignment was performed with BWA [22] and post-alignment modifications (duplicate removal, realignment around INDELs and calibration of the quality scores) were done with the Genome Analysis Toolkit (GATK) [23]. Variant calling was performed with GATK using Variant Quality Score Recalibration (VQSR) method. Putative somatic variants were identified by subtracting the mutations observed in the primary samples from the mutations observed in the corresponding remission samples. SomaticSniper score above 70 was used to identify the final list of somatic events [24].

Variant allele frequency (VAF) plots were drawn for the positions that are novel SNVs in either of the RNA-seq or Exome-seq data and covered by at least 20 reads in both datasets.

### RT-PCR and Sanger sequencing

Novel candidate fusion transcripts were validated by Reverse-Transcription Polymerase-Chain-Reaction (RT-PCR) and Sanger sequencing. In all cases Thymus was used as negative control. cDNA synthesis and PCR amplification were performed using standard protocols that come with Superscript III Reverse Transcriptase (Invitrogen) and GoTaq (Promega). PCR primers were designed to amplify 200–400 bp fragments containing the fusion boundary detected by RNA-seq. The PCR products were analyzed using a QIAxcel automated multicapillary electrophoresis system (QIAGEN). The results were processed and visualized using the BioCalculator Software. PCR products were analyzed by Sanger Sequencing. In cases where multiple PCR products were detected, we performed conventional agarose gel electrophoresis and extraction of specific bands using the gel DNA Recovery Kit (Zymo). Analysis of Sanger chromatograms was performed using CLC Main Workbench 6 (CLC Bio, Aarhus, Denmark). Fusion detection was performed using NCBI Blast alignment. Analysis of the breakpoint was done on the longest isoform reported on the Ensembl genome browser. The tested fusions predictions and the primers used for validations are reported in Table S12.

Validation of *SUZ12* exon skipping was performed by RT-PCR, gel extraction and sequencing of the two PCR products (**Figure 4.I**). The following primers were used for RT-PCR and Sanger sequencing: SUZ12_EX1F (CTGACCACGAGCTTTTCCTC) and SUZ12_EX9R (CCATTTCCTGCATGGCTACT).

### Cloning

The plasmid *TPM3-JAK2* pMSCV-GFP was obtained as follows: a DNA fragment containing *TPM3* coding region till exon 7 was PCR amplified from thymus cDNA using Phusion High Fidelity DNA Polymerase (Finzyme) and primers containing BglII and XhoI restriction sites. Primers containing XhoI and EcoRI restriction sites were used to amplify *JAK2* coding exons 17–25. PCR products were cloned into the BglII and EcoRI sites of the pMSCV-GFP vector after subcloning into the pJET1.2 CloneJET vector (Fermentas). As a final control, plasmid DNA was sequenced by Sanger sequencing.

*SSBP2-FER* fusion was synthesized by Genscript (Piscataway, NJ, USA) and cloned into pMSCV-GFP by using the unique restriction sites XhoI and EcoRI. The plasmid contained the full length *SSBP2-FER* fusion including the first 16 coding exons of *SSBP2* and the coding exons 14–20 of *FER*.

### Cell culture

Viral supernatants were produced in HEK293T cells using an EcoPack packaging plasmid and TurboFect transfection reagent (Fermentas). Viruses were harvested 48 hours after transfection followed by transduction of the Ba/F3 murine pro-B cells (DSMZ, Braunschweig, Germany) as described previously [88].

### Transformation experiments

Ba/F3 cells were washed twice in PBS to remove all traces of cytokines and were seeded in triplicate in 24-well dishes at 100 000 cells/mL. GFP expression and cell number were measure on a Guava flow cytometer (Millipore). All experiments were terminated at day 8 after cytokine removal and cell lines showing no sign of cell proliferation at that timepoint were declared to be non-transforming.

### Western blotting

Total cell lysates were analyzed by standard electrophoresis and western blotting procedures using the following antibodies: anti-phospho-*JAK1* (Tyr1022/1023), anti-phospho-*STAT1*, anti–phospho-*STAT5* (Tyr694), anti–phospho-*STAT3* (Tyr705), anti-phospho*ERK1*-2, anti-phospho-SRC families (Tyr416) (from Cell Signaling Technology).

### Inhibitor experiments

*TPM3-JAK2* and *SSBP2-FER* IL3-independent Ba/F3 cells were seeded in triplicate in 96-well plates at a density of 0.03×10^6^ cells in the presence of *JAK* inhibitor Ruxolitinib (INCB018424, Azon Medchem). Cell proliferation and viability were assessed on a Guava flow cytometer after 24 hours to determine the IC50, the concentration of inhibitor that gave a 50% inhibition.

### Accession numbers

Genome data has been deposited at the European Genome-phenome Archive (EGA, http://www.ebi.ac.uk/ega/) which is hosted at the EBI, under accession number EGAS00001000536.

## Supporting Information

Figure S1 Suboptimal mapping strategies result in incorrect read alignment. Alignment of the Exome-seq and RNA-seq reads on *GLUD2* and *GLUD1* genes for the RPMI8402 cell line. Two alignment strategies are visualized in these figures for RNA-seq: genome-only mapping and combined mapping strategy. Panel (**A**) shows the alignment for *GLUD2* gene. With exome-seq a very high coverage was achieved (the coverage track scale is 0–1000). Aligning the RNA-seq reads with ‘genome-only’ option yields high coverage as well however with a lot of mismatches in the alignment (colored lines indicate the presence of a nucleotide different than the reference base). However, when combined mapping strategy is applied the coverage drops drastically. Panel (**B**) shows the alignment of *GLUD1* gene. When mapping with genome only option, the coverage is not high (the coverage track scale is 0–900) since the reads are forced to map to the pseudogene (*GLUD2*) with a lot of mismatched. When the combined mapping strategy implemented, the reads align to *GLUD1* gene correctly with less mismatches.
(PDF)

Figure S2 Variant allele frequency plots for assessing transcriptome-only mapping strategy. The variant allele frequencies of the SNVs that have at least 20× reads in exome-seq and RNA-seq are plotted. The RNA-seq SNVs were obtained with the transcriptome-only alignment option. Red and green dots represent the SNVs that are detected only in RNA-seq and only in exome-seq, respectively, while black dots represent the SNVs that are called in both. Venn diagrams are produced from the points represented in the graphs. The plots are generated for (**A**) RPMI8402 cell line and (**B**) TLE79 patient sample.
(PDF)

Figure S3 Variant Allele Frequency (VAF) plots for 16 cell lines and 20 patient samples. RNA-seq calls are made with combined mapping strategy. The venn diagrams and VAF plots are drawn for variants that have sequence coverage of at least 20×.
(PDF)

Figure S4 Scatter plot of average coverage versus recall ratio per sample. Recall ratio per sample is calculated as the percentage of Exome-seq SNVs that are called in the RNA-seq as well. Recall ratio 0.3 is assumed as the indicator of a ‘good sample’ in terms of variant detection.
(PDF)

Figure S5 Visualization of the alignments with Exome-seq and RNA-seq for the 5 INDELs that are validated in the DNA of the samples but absent in the RNA-seq alignments. The Exome-seq and RNA-seq alignment files are visualized using IGV for (**A**) *KDM6A* in TLE87, (**B**) *PTEN* in TLE92, (**C**) *WT1* in TLE76, (**D**) *USP9X* in SUPT1, and (**E**) *UNC5D* in MOLT4. The exome-seq alignment files (below) have the reads containing the INDEL, whereas RNA-seq alignment files (above) either contain reads with reference only (A, B, and E) or a small portion of reads with INDEL (C and D).
(PDF)

Figure S6 INDELs in TLE92 and TLE87 are detected after mapping with a different aligner. The screenshots from UCSC genome browser shows (**A**) the 4 bp deletion in *PTEN* (note that only a part of the alignment was shown) and (**B**) 1 bp deletion in *KDM6A*. In both cases BWA transcriptome-only mapping was coupled to BLAT genome mapping. In (**C**) and (**D**), TopHat2 transcriptome-only mapping coupled with BLAT genome mapping was displayed for *PTEN* and KMD6A *INDELs*, respectively.
(PDF)

Figure S7 Batch effect removal for gene expression profiling. Multidimensional scaling (MDS) plots before and after batch effect removal. A batch effect was observed whereby samples originating from the same collection center clustered together based on the edgeR normalized gene-by-gene counts (**A**). A similar clustering was observed when the FPKM values per transcript was used (**B**). After fitting a Generalized Linear Model (on the edgeR normalized gene-by-gene counts) accounting for sample collection center, the aberrant clustering of the samples is corrected (**C**).
(PDF)

Figure S8 Overview of exon skipping event in *LCK*. (**A**) Predicted novel transcript of *LCK* aligned with known *LCK* isoforms. Dotted red box indicates the exon-skipping event in the 8th exon (**B**) Sashimi plot detailing the junction supporting the exon skipping event in patient samples R5, R5 and TLE93 with respect to Thymus. (**C**) Schematic representation of the predicted alternative splicing event of *LCK*. The exon skipping ratio (C/A+B+C) of exon 8 of *LCK* in R5, R4, TLE93 are 0.40, 0.47 and 0.20, respectively. (**D**) Schematic overview of *LCK* protein illustrating the spliced out portion without affecting the functional domains.
(PDF)

Figure S9 Schematic overview of the SUZ12 exon-skipping event. (**A**) Schematic representation of the predicted alternative splicing event of *SUZ12*. The exon skipping ratio (C/A+B+C) of exon 7 of *SUZ12* in R5 is 0.35. (**B**) Schematic overview of SUZ12 protein illustrating the spliced out portion without affecting the functional domains.
(PDF)

Figure S10 Out-of-frame fusions can have various consequences. The over or under expression caused by out-of-frame gene fusions are illustrated in the normalized expression heatmap. *CLINT1-MEF2C, HNRP-ZNF219, ZEB1-BMI1* and *AHI1-MYB* fusion are associated with overexpression of *MEF2C, ZNF219, BMI1* and *MYB*; whereas as *TP53-TBC1D3F, PTEN-RNLS, IKZF1-ABCA13* and *CDKN2A-miR31HG* fusions are responsible for the under-expression of *TP53, PTEN, IKZF1* and *CDKN2A*.
(PDF)

Table S1 (**A**) Sequencing and mapping statistics, (**B**) Variant statistics, (**C**) Fusion statistics.
(XLSX)

Table S2 Samples analyzed in this study.
(XLSX)

Table S3 Comparison of the number of novel SNV and INDELs between RNAseq and Exome-seq.
(XLSX)

Table S4 Validated INDELs from the Exome-seq.
(XLSX)

Table S5 Mutations detected in 213 genes.
(XLSX)

Table S6 IPA on 213 candidate genes.
(XLSX)

Table S7 ENDEAVOUR results on 213 genes.
(XLSX)

Table S8 ATEs identified in known T-ALL drivers.
(XLSX)

Table S9 Fusions detected in 49 samples and the Thymus.
(XLSX)

Table S10 Annotation of fusions with Pegasus.
(XLSX)

Table S11 Patient characteristics.
(XLSX)

Table S12 Novel Fusion Transcript validated by RT-PCR and Sanger sequencing.
(XLSX)
