# Supplementary material for: Comprehensive Analysis of Transcriptome Variation Uncovers Known and Novel Driver Events in T-Cell Acute Lymphoblastic Leukemia
Source: PLoS Genet. 2013 Dec 19;9(12):e1003997. doi: 10.1371/journal.pgen.1003997 (PMC3868543; doi:10.1371/journal.pgen.1003997)
Supplement: Figure S1 — Suboptimal mapping strategies result in incorrect read alignment. Alignment of the Exome-seq and RNA-seq reads on GLUD2 and GLUD1 genes for the RPMI8402 cell line. Two alignment strategies are visualized in these figures for RNA-seq: genome-only mapping and combined mapping strategy. Panel (A) shows the alignment for GLUD2 gene. With exome-seq a very high coverage was achieved (the coverage track scale is 0–1000). Aligning the RNA-seq reads with ‘genome-only’ option yields high coverage as well however with a lot of mismatches in the alignment (colored lines indicate the presence of a nucleotide different than the reference base). However, when combined mapping strategy is applied the coverage drops drastically. Panel (B) shows the alignment of GLUD1 gene. When mapping with genome only option, the coverage is not high (the coverage track scale is 0–900) since the reads are forced to map to the pseudogene (GLUD2) with a lot of mismatched. When the combined mapping strategy implemented, the reads align to GLUD1 gene correctly with less mismatches. (PDF) [file pgen.1003997.s001.pdf]

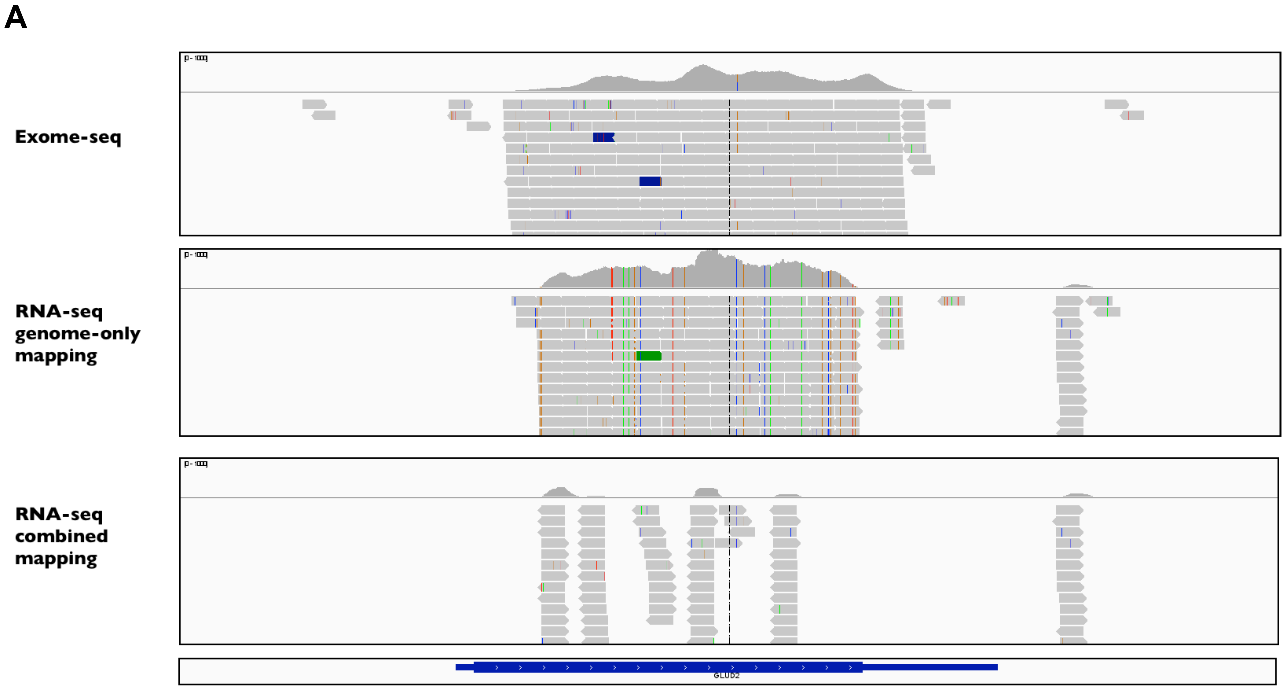
A
Exome-seq
RNA-seq genome-only mapping
RNA-seq combined mapping
GLUD2

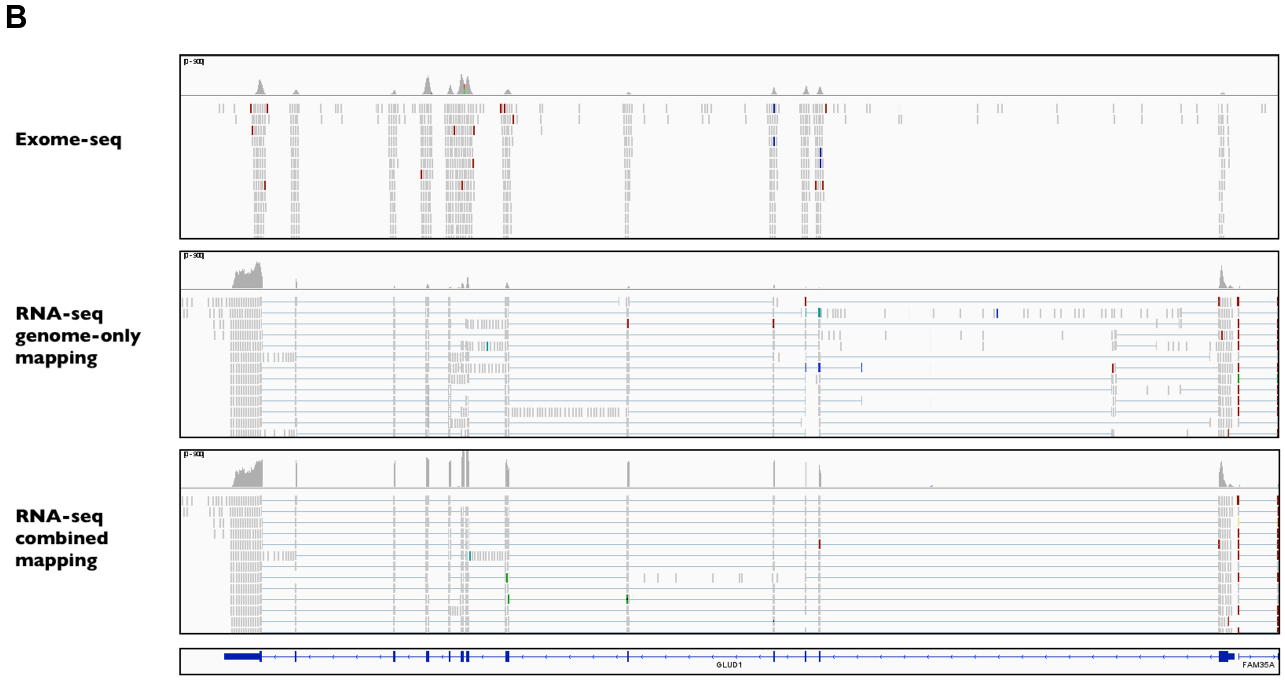
B
Exome-seq
RNA-seq genome-only mapping
RNA-seq combined mapping
GLUD1
FAM35A
