# Supplementary figures and images for: Comprehensive Analysis of Transcriptome Variation Uncovers Known and Novel Driver Events in T-Cell Acute Lymphoblastic Leukemia
Source: PLoS Genet. 2013 Dec 19;9(12):e1003997. doi: 10.1371/journal.pgen.1003997 (PMC3868543; doi:10.1371/journal.pgen.1003997)

**A**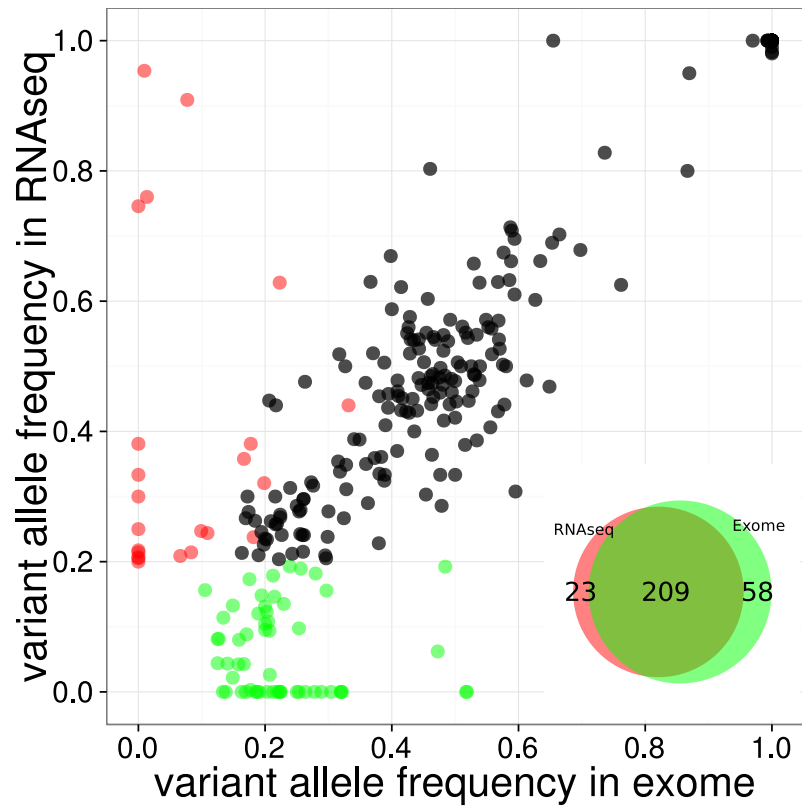**B**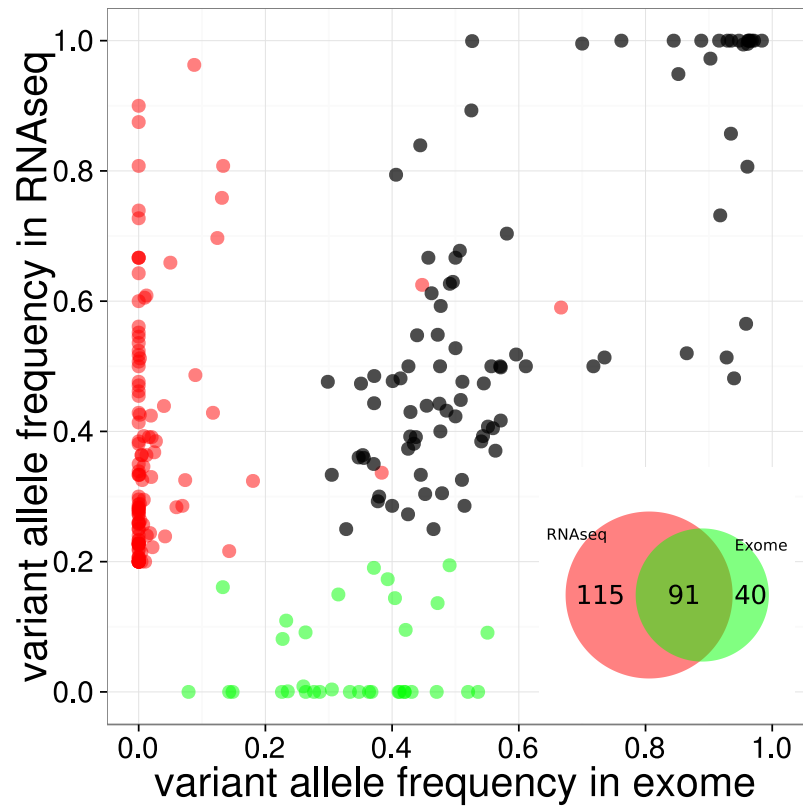

Supplement: Figure S2 — Variant allele frequency plots for assessing transcriptome-only mapping strategy. The variant allele frequencies of the SNVs that have at least 20× reads in exome-seq and RNA-seq are plotted. The RNA-seq SNVs were obtained with the transcriptome-only alignment option. Red and green dots represent the SNVs that are detected only in RNA-seq and only in exome-seq, respectively, while black dots represent the SNVs that are called in both. Venn diagrams are produced from the points represented in the graphs. The plots are generated for (A) RPMI8402 cell line and (B) TLE79 patient sample. (PDF) [file pgen.1003997.s002.pdf]

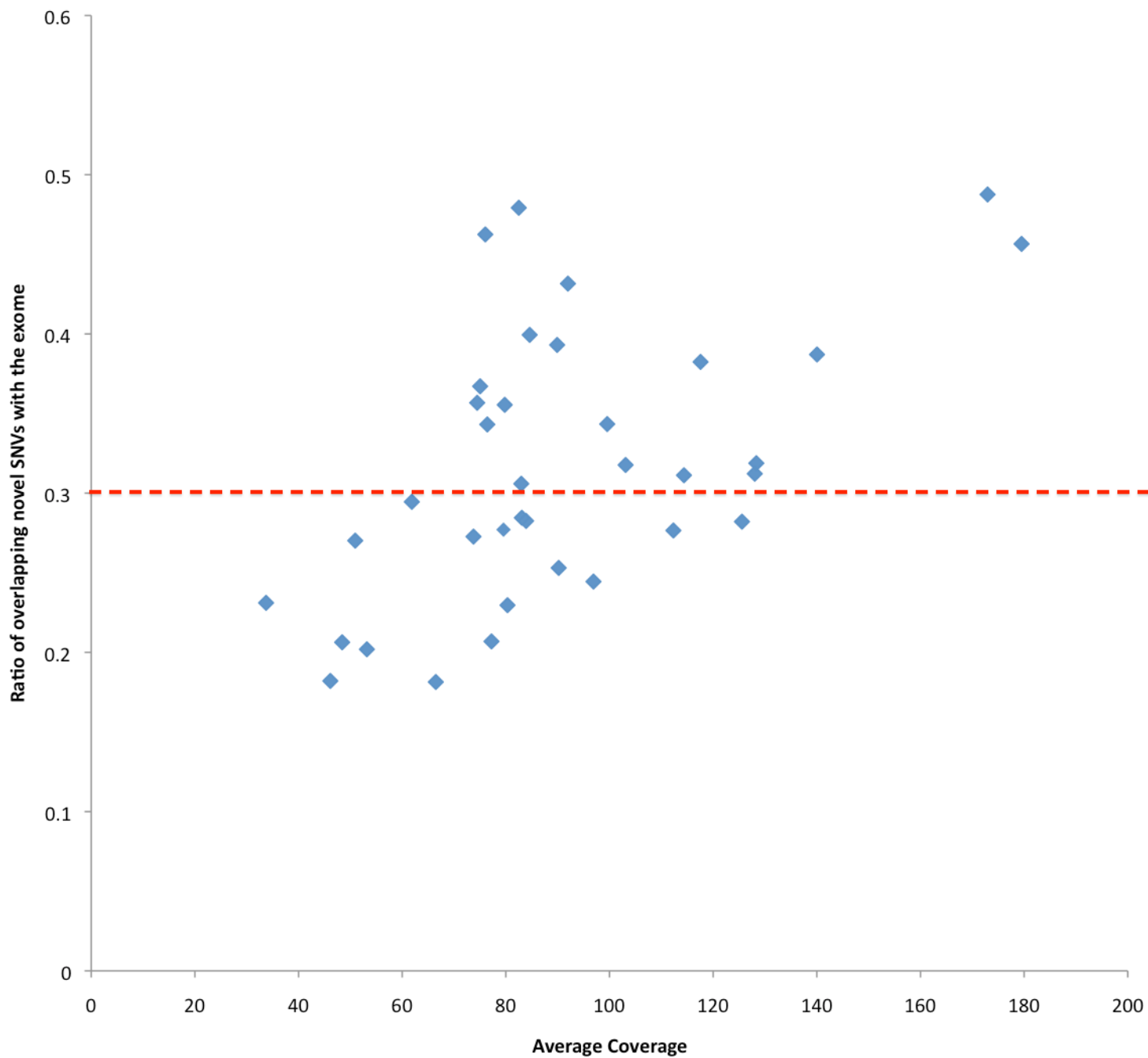

Supplement: Figure S4 — Scatter plot of average coverage versus recall ratio per sample. Recall ratio per sample is calculated as the percentage of Exome-seq SNVs that are called in the RNA-seq as well. Recall ratio 0.3 is assumed as the indicator of a ‘good sample’ in terms of variant detection. (PDF) [file pgen.1003997.s004.pdf]

**A**

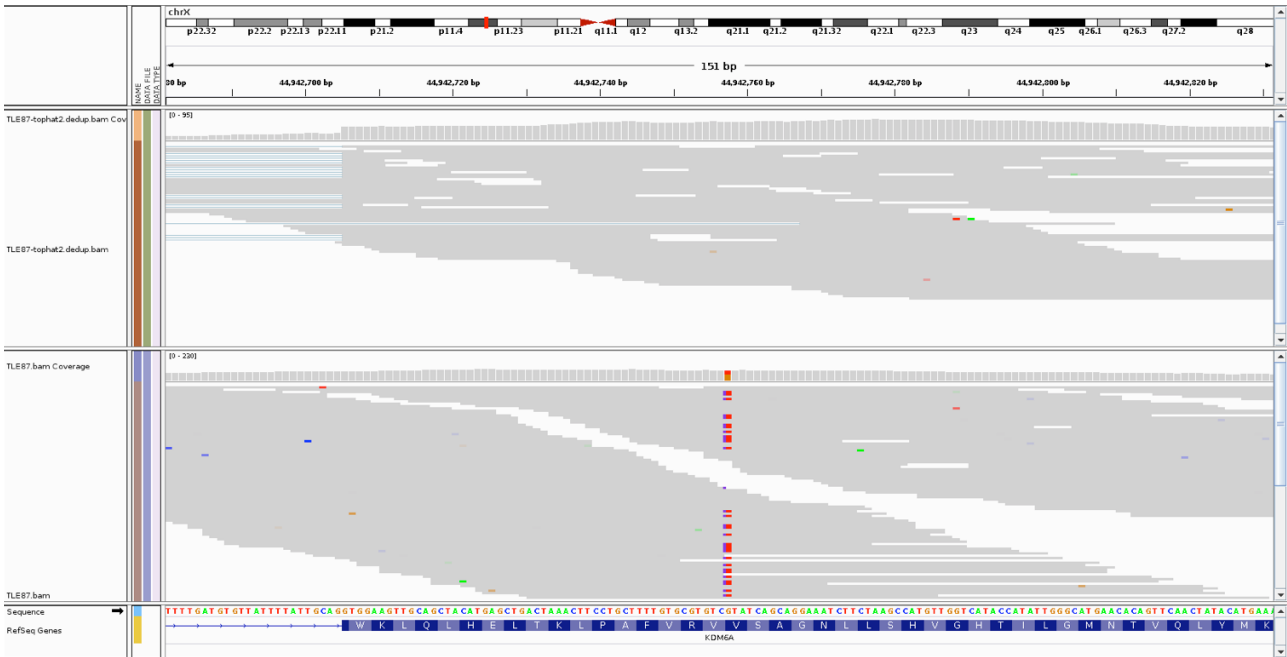

**B**

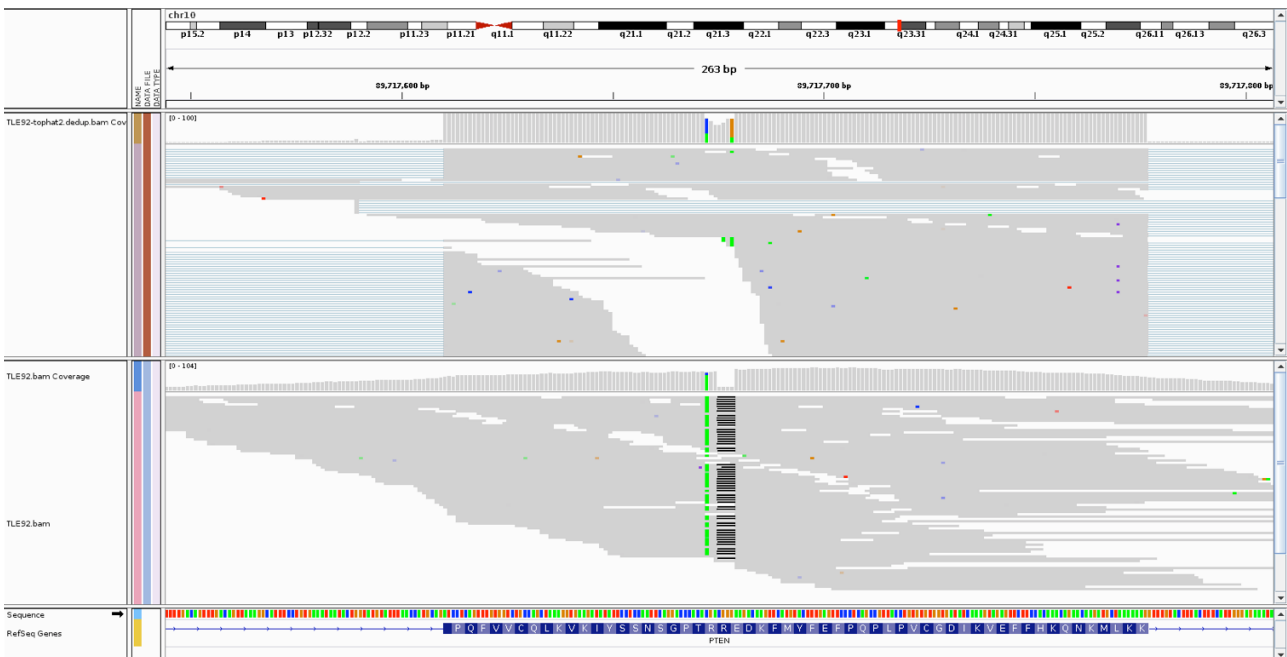

C

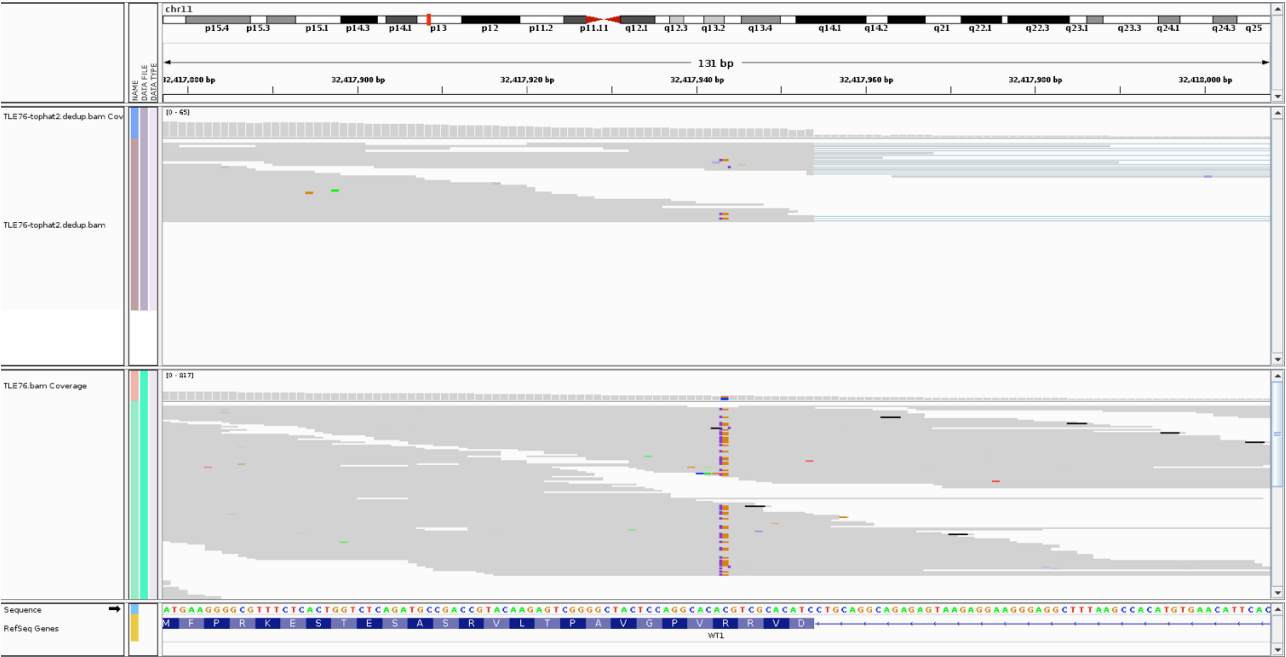

D

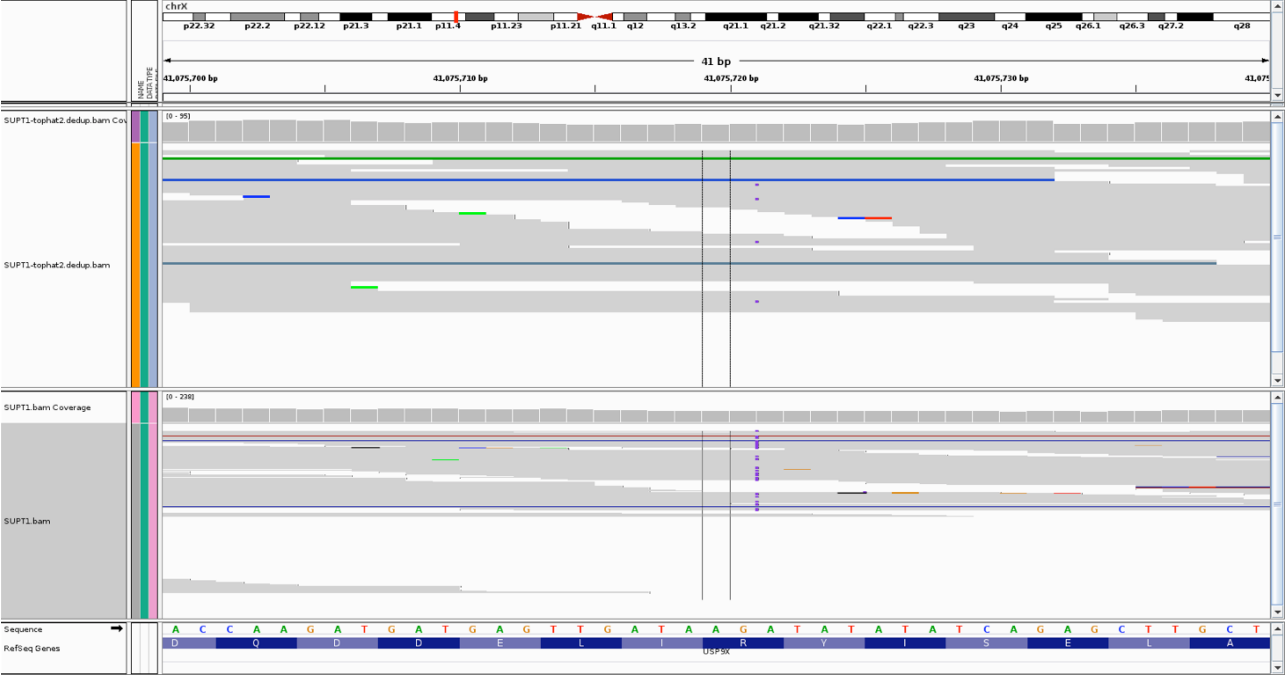

E

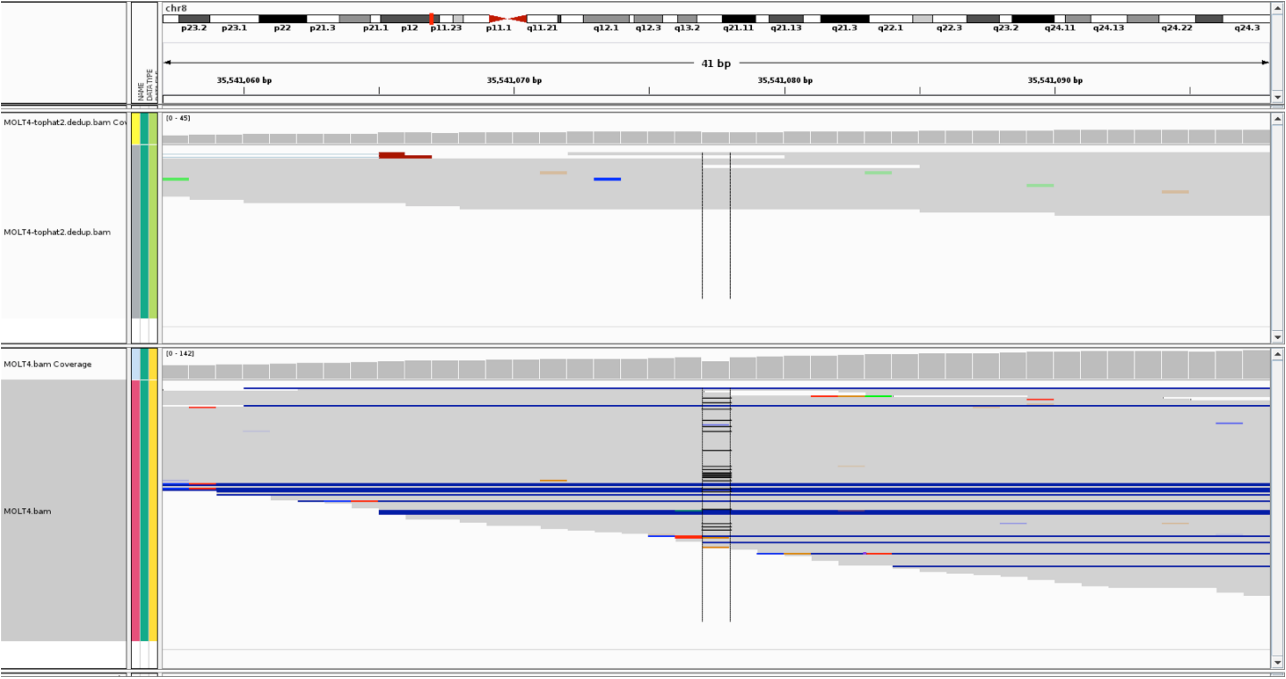

Supplement: Figure S5 — Visualization of the alignments with Exome-seq and RNA-seq for the 5 INDELs that are validated in the DNA of the samples but absent in the RNA-seq alignments. The Exome-seq and RNA-seq alignment files are visualized using IGV for (A) KDM6A in TLE87, (B) PTEN in TLE92, (C) WT1 in TLE76, (D) USP9X in SUPT1, and (E) UNC5D in MOLT4. The exome-seq alignment files (below) have the reads containing the INDEL, whereas RNA-seq alignment files (above) either contain reads with reference only (A, B, and E) or a small portion of reads with INDEL (C and D). (PDF) [file pgen.1003997.s005.pdf]

**A**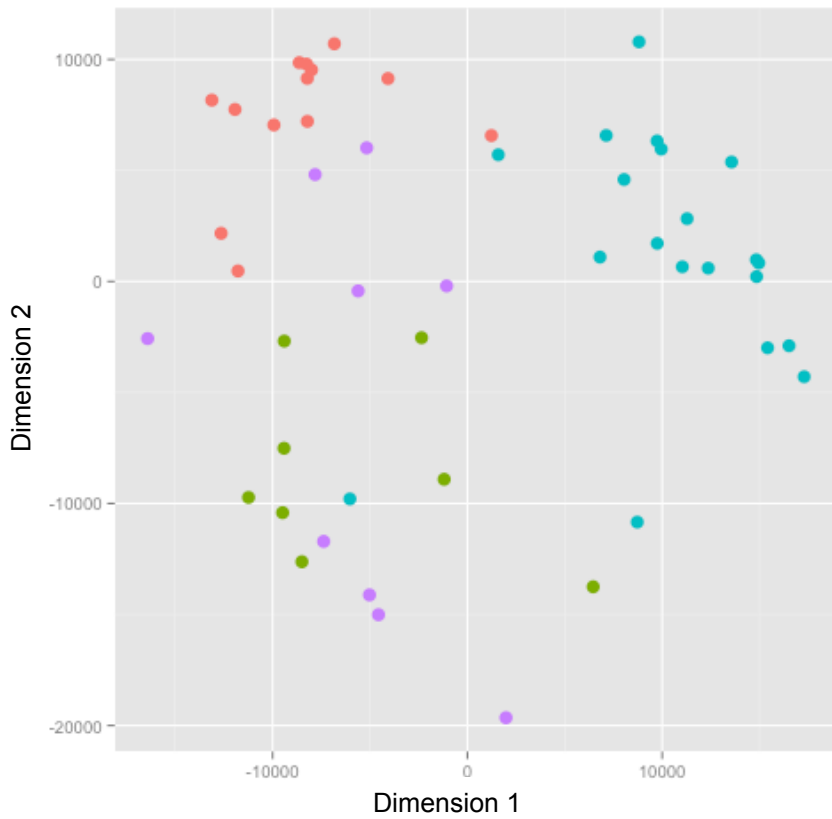**B**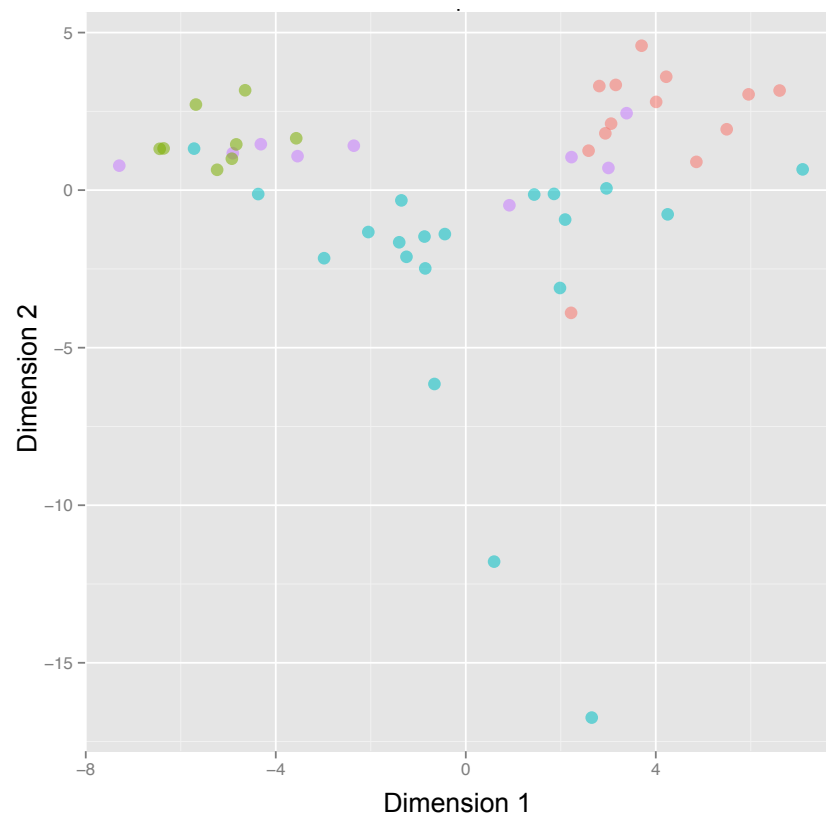**C**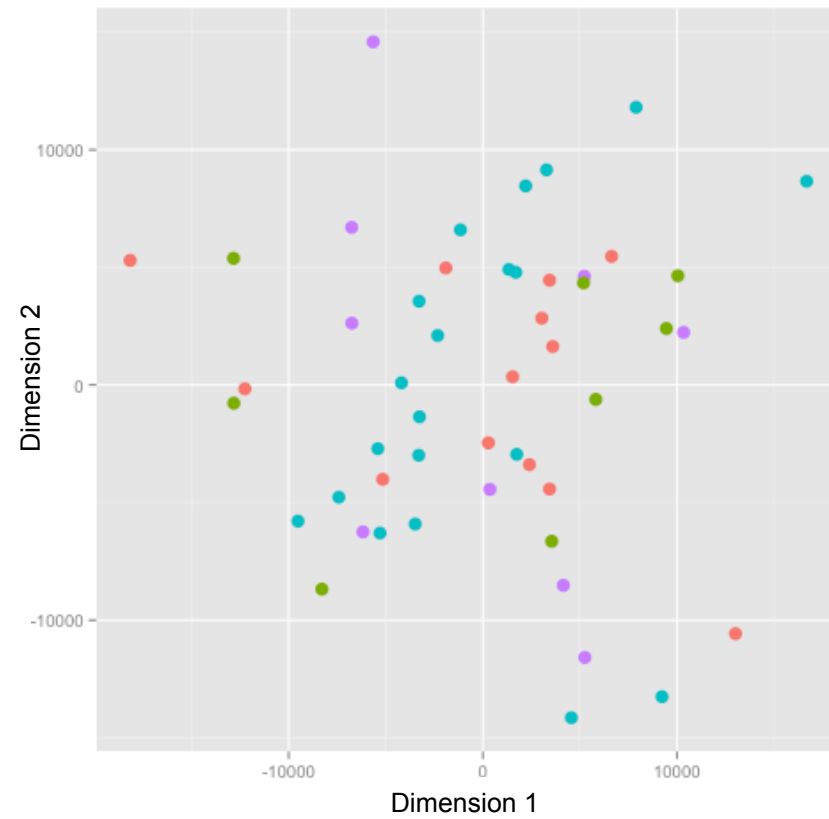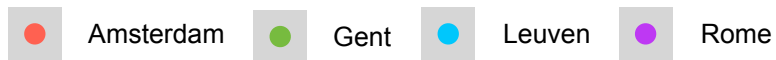

Supplement: Figure S7 — Batch effect removal for gene expression profiling. Multidimensional scaling (MDS) plots before and after batch effect removal. A batch effect was observed whereby samples originating from the same collection center clustered together based on the edgeR normalized gene-by-gene counts (A). A similar clustering was observed when the FPKM values per transcript was used (B). After fitting a Generalized Linear Model (on the edgeR normalized gene-by-gene counts) accounting for sample collection center, the aberrant clustering of the samples is corrected (C). (PDF) [file pgen.1003997.s007.pdf]

# A

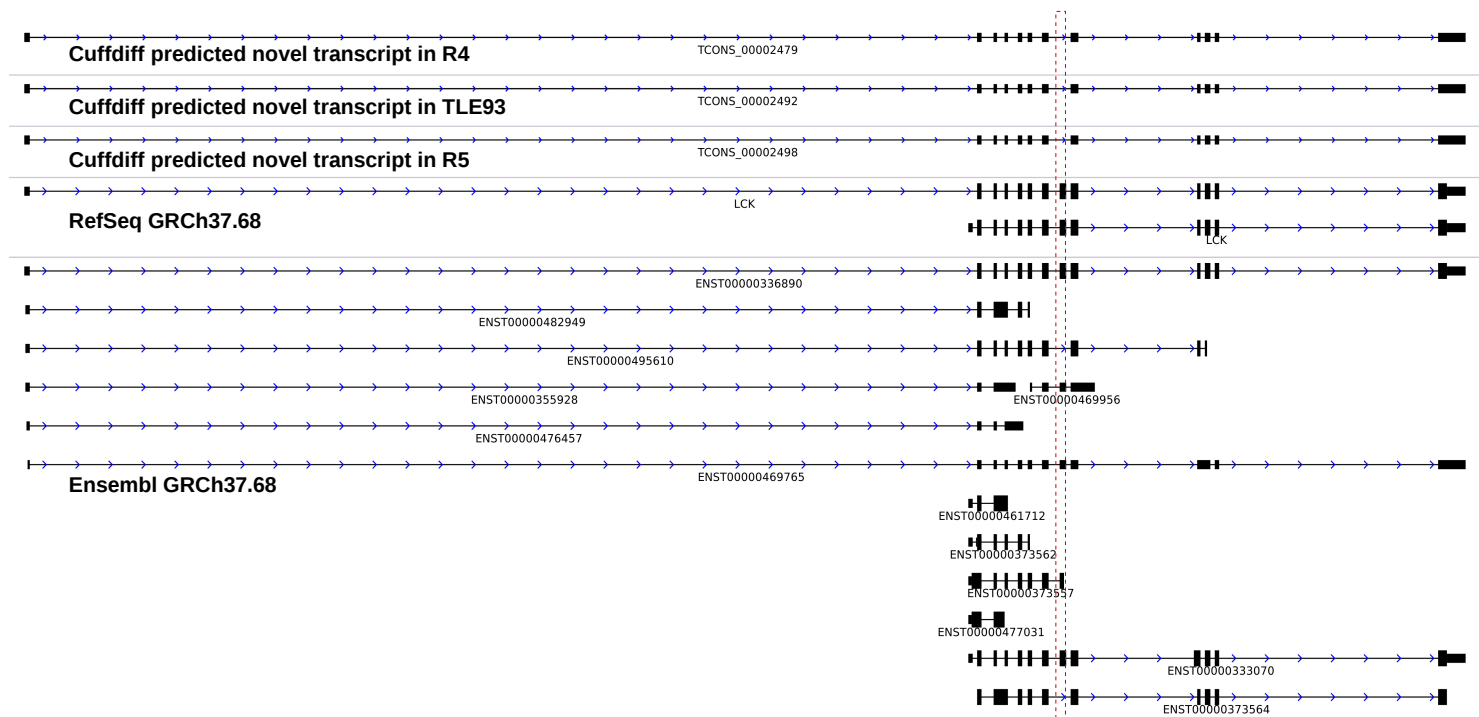

# B

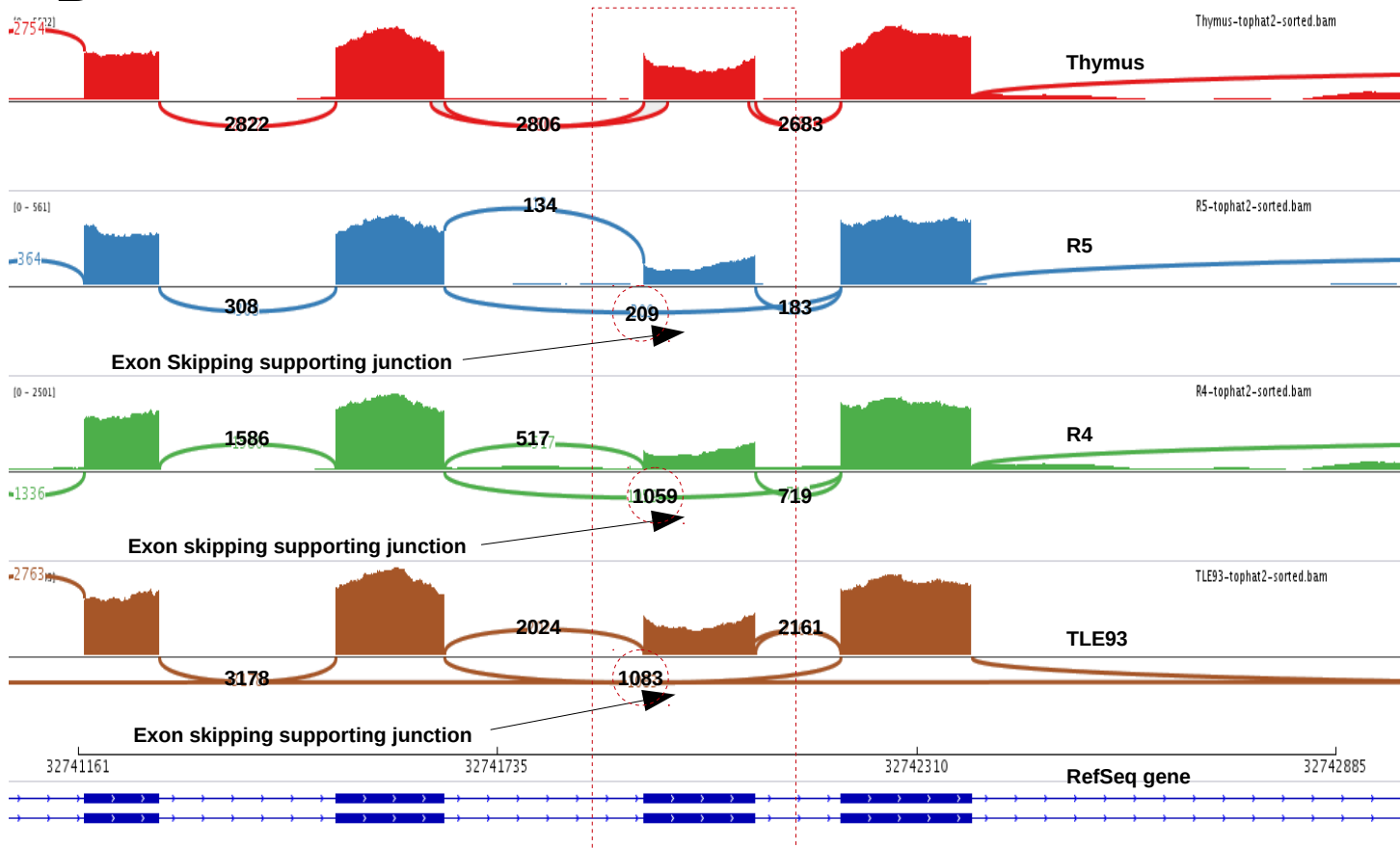

**C**

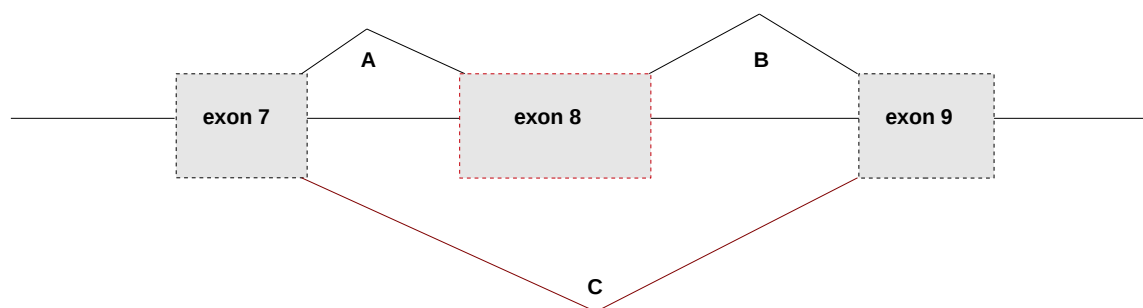

**D**

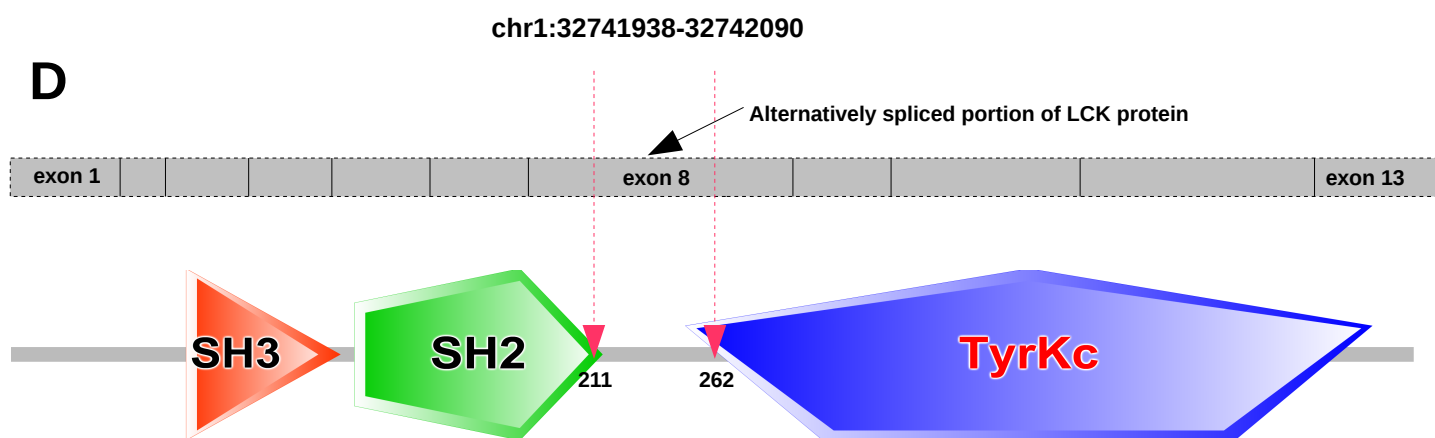

Supplement: Figure S8 — Overview of exon skipping event in LCK. (A) Predicted novel transcript of LCK aligned with known LCK isoforms. Dotted red box indicates the exon-skipping event in the 8th exon (B) Sashimi plot detailing the junction supporting the exon skipping event in patient samples R5, R5 and TLE93 with respect to Thymus. (C) Schematic representation of the predicted alternative splicing event of LCK. The exon skipping ratio (C/A+B+C) of exon 8 of LCK in R5, R4, TLE93 are 0.40, 0.47 and 0.20, respectively. (D) Schematic overview of LCK protein illustrating the spliced out portion without affecting the functional domains. (PDF) [file pgen.1003997.s008.pdf]

**A**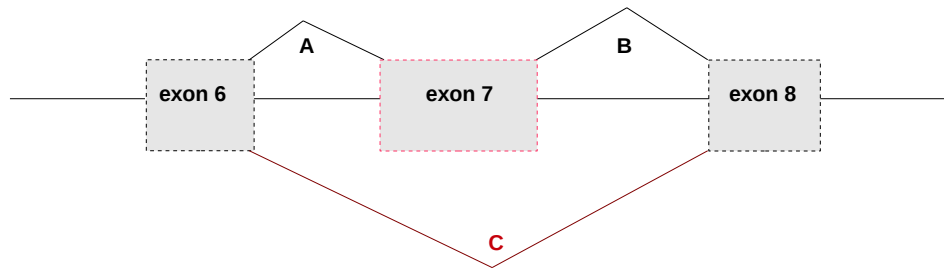**B**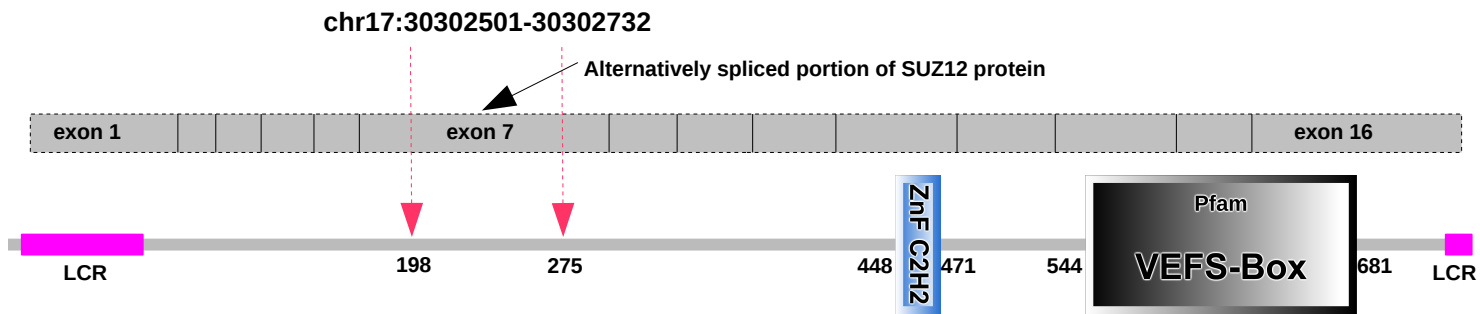

Supplement: Figure S9 — Schematic overview of the SUZ12 exon-skipping event. (A) Schematic representation of the predicted alternative splicing event of SUZ12. The exon skipping ratio (C/A+B+C) of exon 7 of SUZ12 in R5 is 0.35. (B) Schematic overview of SUZ12 protein illustrating the spliced out portion without affecting the functional domains. (PDF) [file pgen.1003997.s009.pdf]

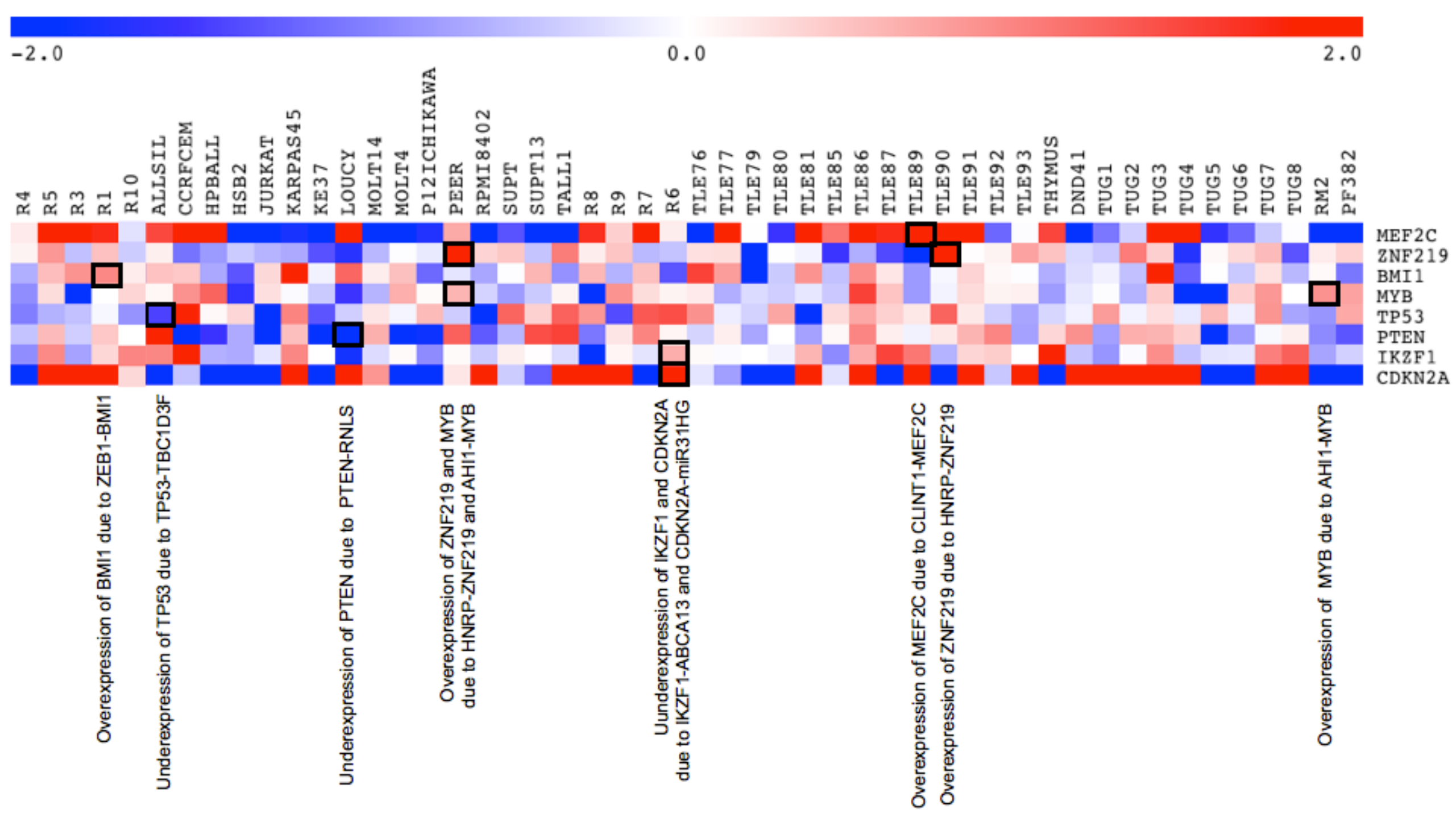

Supplement: Figure S10 — Out-of-frame fusions can have various consequences. The over or under expression caused by out-of-frame gene fusions are illustrated in the normalized expression heatmap. CLINT1-MEF2C, HNRP-ZNF219, ZEB1-BMI1 and AHI1-MYB fusion are associated with overexpression of MEF2C, ZNF219, BMI1 and MYB; whereas as TP53-TBC1D3F, PTEN-RNLS, IKZF1-ABCA13 and CDKN2A-miR31HG fusions are responsible for the under-expression of TP53, PTEN, IKZF1 and CDKN2A. (PDF) [file pgen.1003997.s010.pdf]
