# Supplementary material for: Comprehensive Analysis of Transcriptome Variation Uncovers Known and Novel Driver Events in T-Cell Acute Lymphoblastic Leukemia
Source: PLoS Genet. 2013 Dec 19;9(12):e1003997. doi: 10.1371/journal.pgen.1003997 (PMC3868543; doi:10.1371/journal.pgen.1003997)
Supplement: Figure S3 — Variant Allele Frequency (VAF) plots for 16 cell lines and 20 patient samples. RNA-seq calls are made with combined mapping strategy. The venn diagrams and VAF plots are drawn for variants that have sequence coverage of at least 20×. (PDF) [file pgen.1003997.s003.pdf]

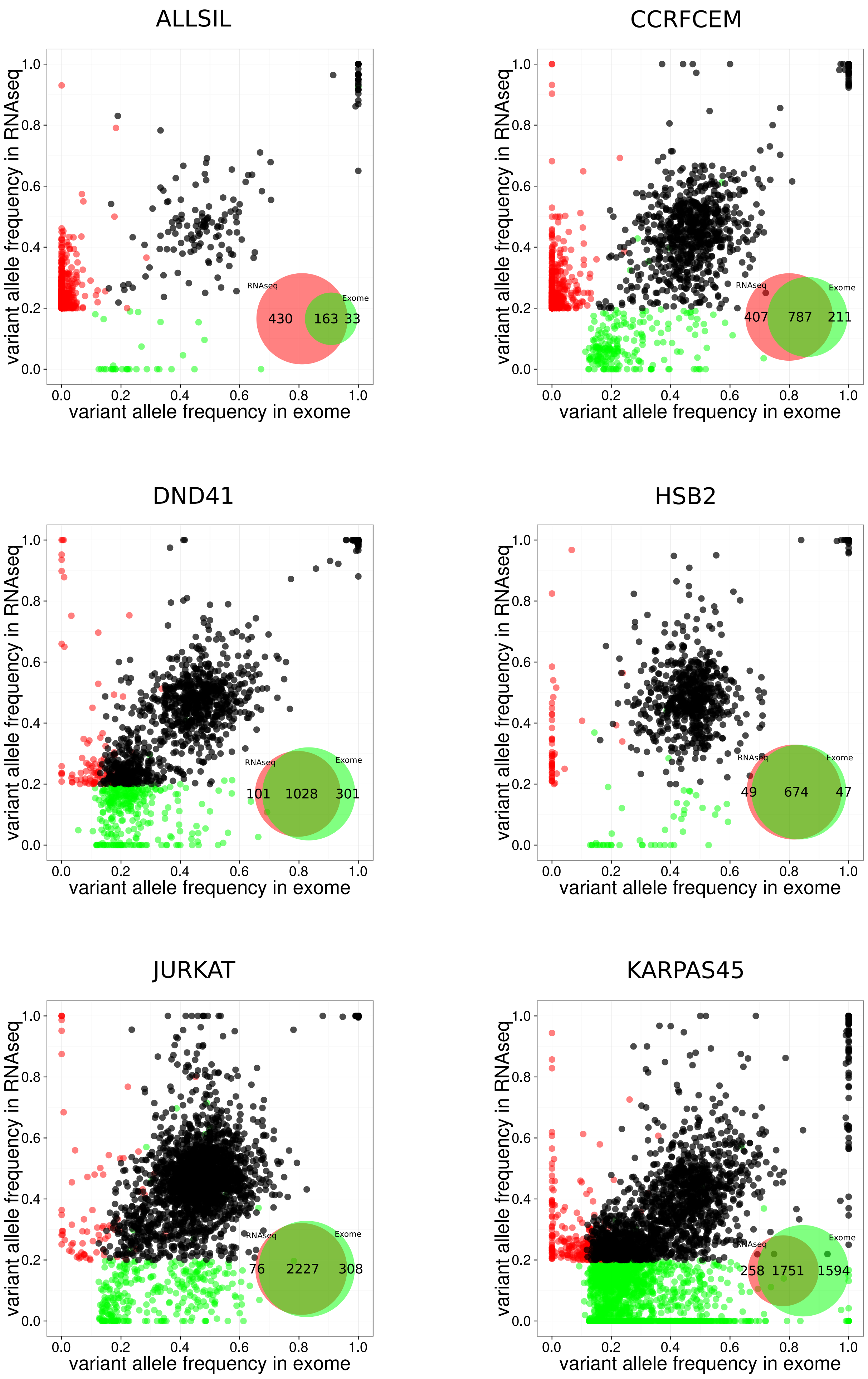
ALLSIL
430
163
33
CCRFCEM
407
787
211
DND41
101
1028
301
HSB2
49
674
47
JURKAT
76
2227
308
KARPAS45
258
1751
1594
RNAseq
Exome
variant allele frequency in RNAseq
variant allele frequency in exome
0.0
0.2
0.4
0.6
0.8
1.0

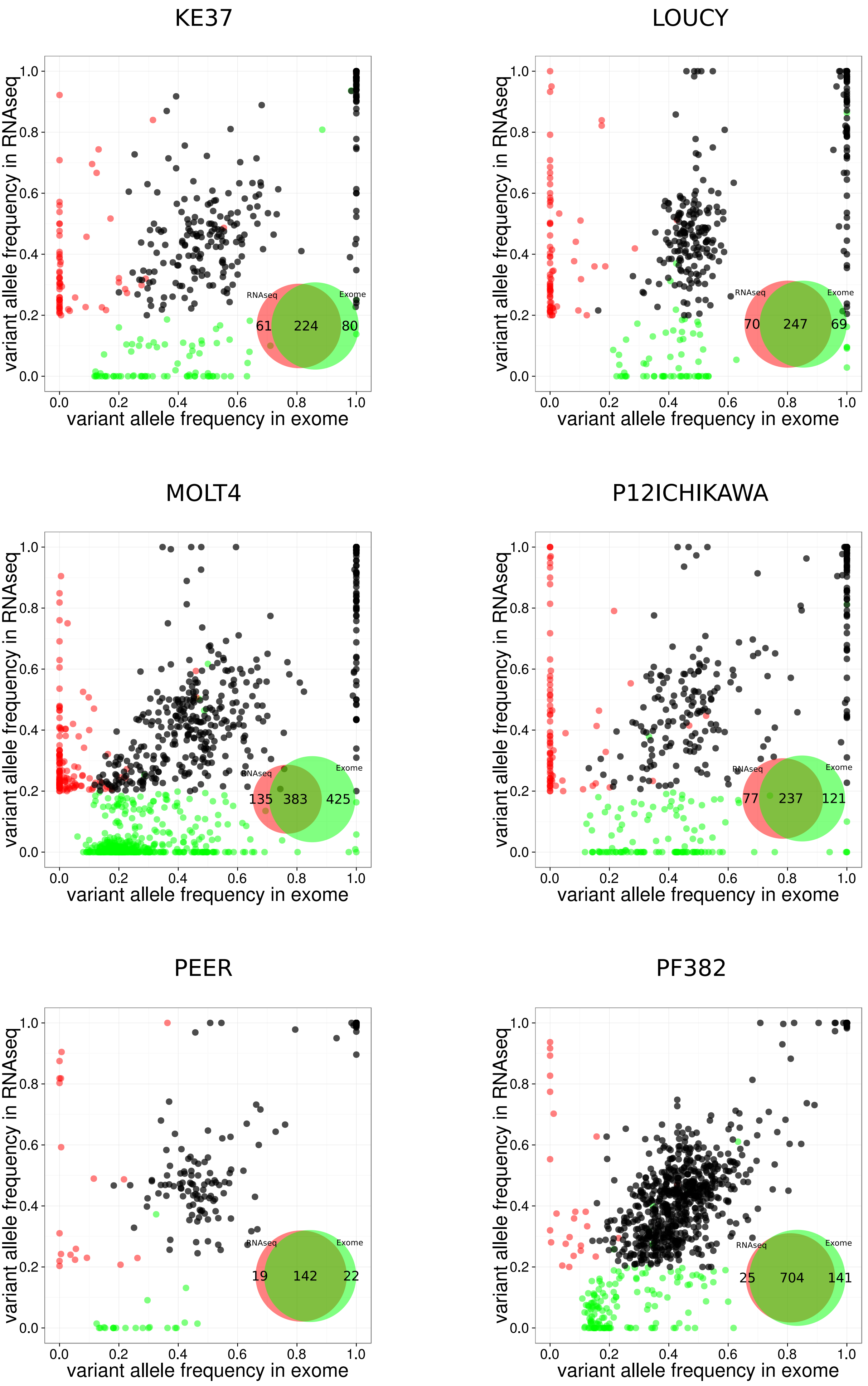
KE37
variant allele frequency in RNAseq
variant allele frequency in exome
RNAseq
Exome
61
224
80
LOUCY
variant allele frequency in RNAseq
variant allele frequency in exome
RNAseq
Exome
70
247
69
MOLT4
variant allele frequency in RNAseq
variant allele frequency in exome
RNAseq
Exome
135
383
425
P12ICHIKAWA
variant allele frequency in RNAseq
variant allele frequency in exome
RNAseq
Exome
77
237
121
PEER
variant allele frequency in RNAseq
variant allele frequency in exome
RNAseq
Exome
19
142
22
PF382
variant allele frequency in RNAseq
variant allele frequency in exome
RNAseq
Exome
25
704
141

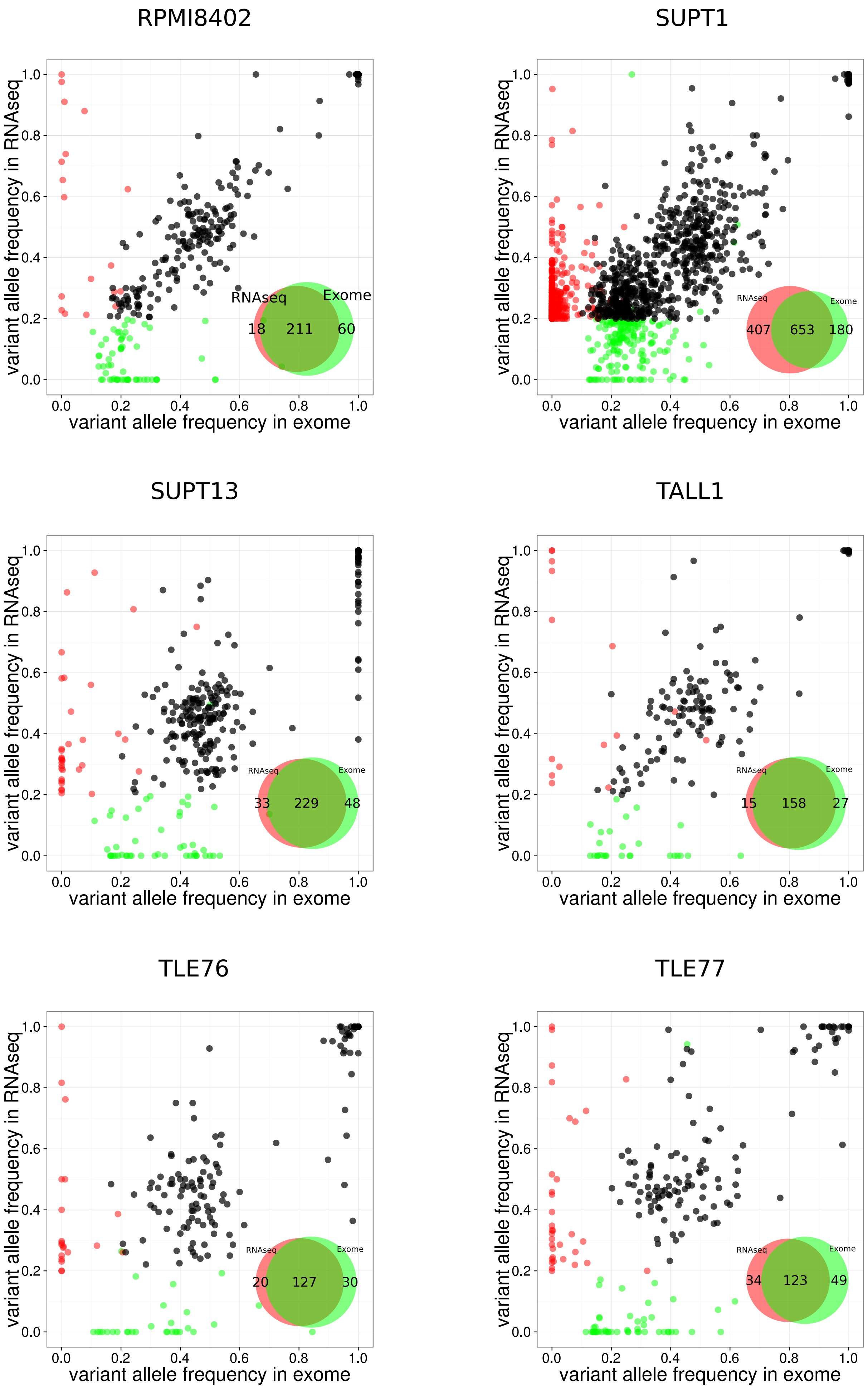
RPMI8402
variant allele frequency in RNAseq
variant allele frequency in exome
RNAseq
Exome
18
211
60
SUPT1
variant allele frequency in RNAseq
variant allele frequency in exome
RNAseq
Exome
407
653
180
SUPT13
variant allele frequency in RNAseq
variant allele frequency in exome
RNAseq
Exome
33
229
48
TALL1
variant allele frequency in RNAseq
variant allele frequency in exome
RNAseq
Exome
15
158
27
TLE76
variant allele frequency in RNAseq
variant allele frequency in exome
RNAseq
Exome
20
127
30
TLE77
variant allele frequency in RNAseq
variant allele frequency in exome
RNAseq
Exome
34
123
49

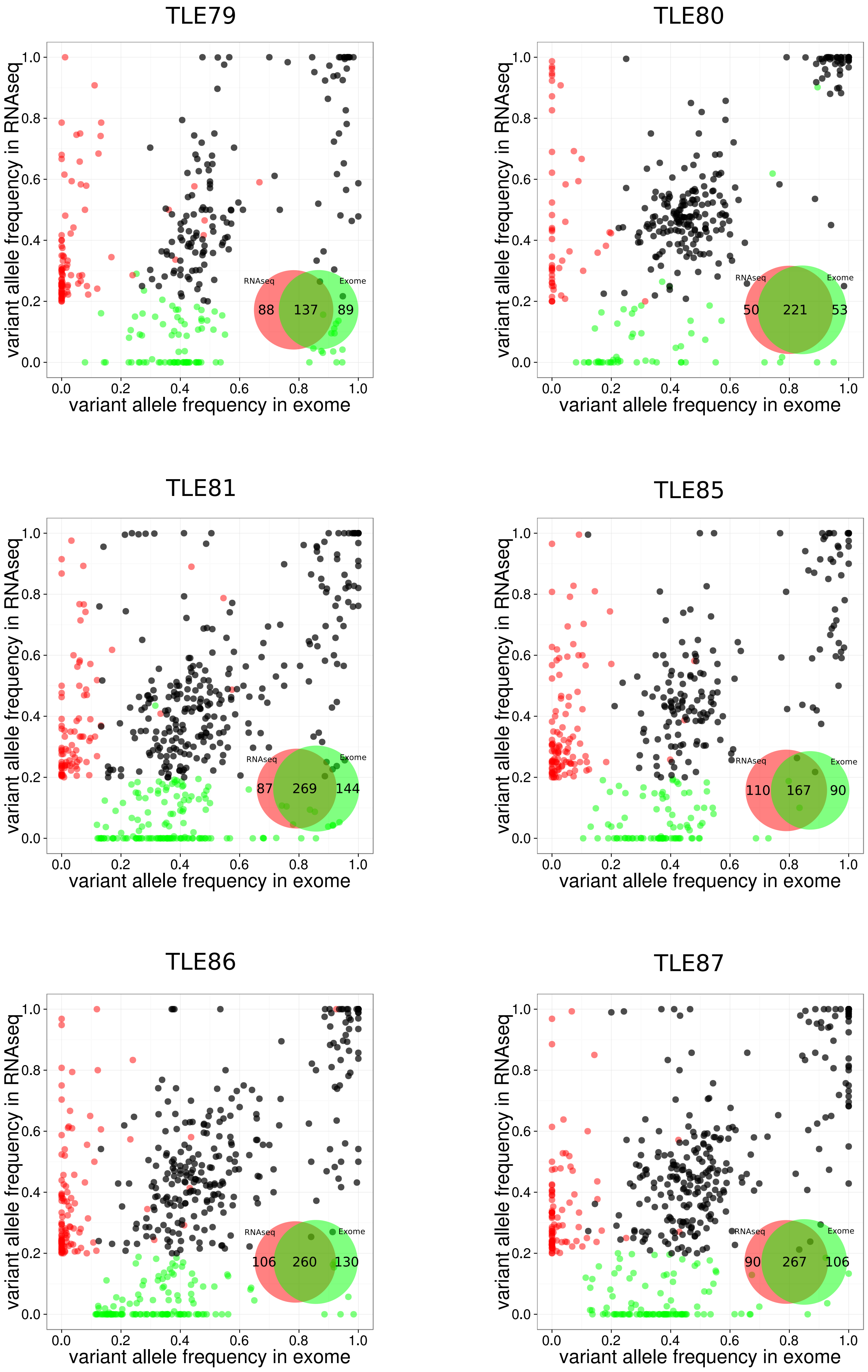
TLE79
88
137
89
TLE80
50
221
53
TLE81
87
269
144
TLE85
110
167
90
TLE86
106
260
130
TLE87
90
267
106
RNAseq
Exome
variant allele frequency in RNAseq
variant allele frequency in exome

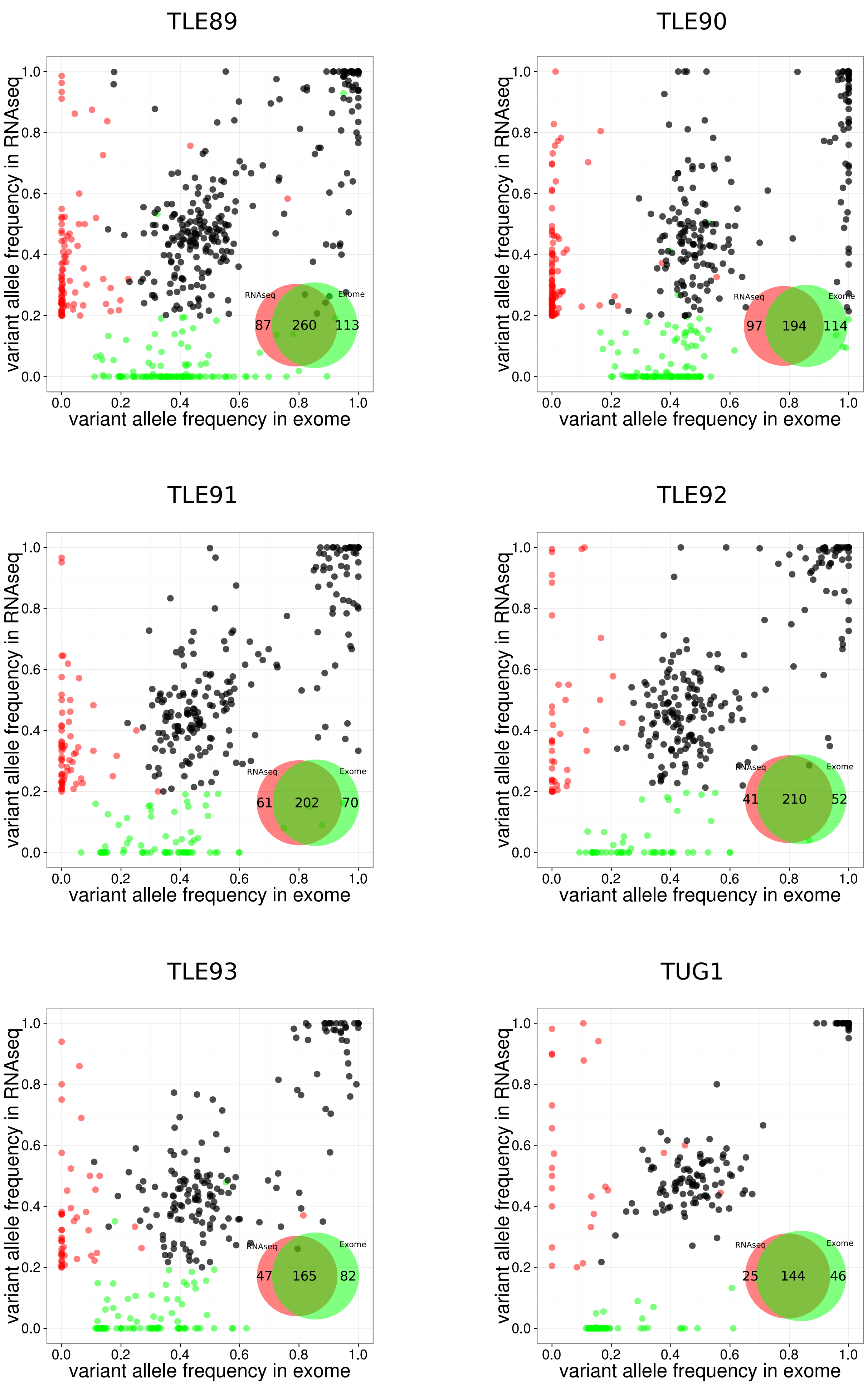
TLE89
variant allele frequency in RNAseq
variant allele frequency in exome
RNAseq
Exome
87
260
113
TLE90
variant allele frequency in RNAseq
variant allele frequency in exome
RNAseq
Exome
97
194
114
TLE91
variant allele frequency in RNAseq
variant allele frequency in exome
RNAseq
Exome
61
202
70
TLE92
variant allele frequency in RNAseq
variant allele frequency in exome
RNAseq
Exome
41
210
52
TLE93
variant allele frequency in RNAseq
variant allele frequency in exome
RNAseq
Exome
47
165
82
TUG1
variant allele frequency in RNAseq
variant allele frequency in exome
RNAseq
Exome
25
144
46

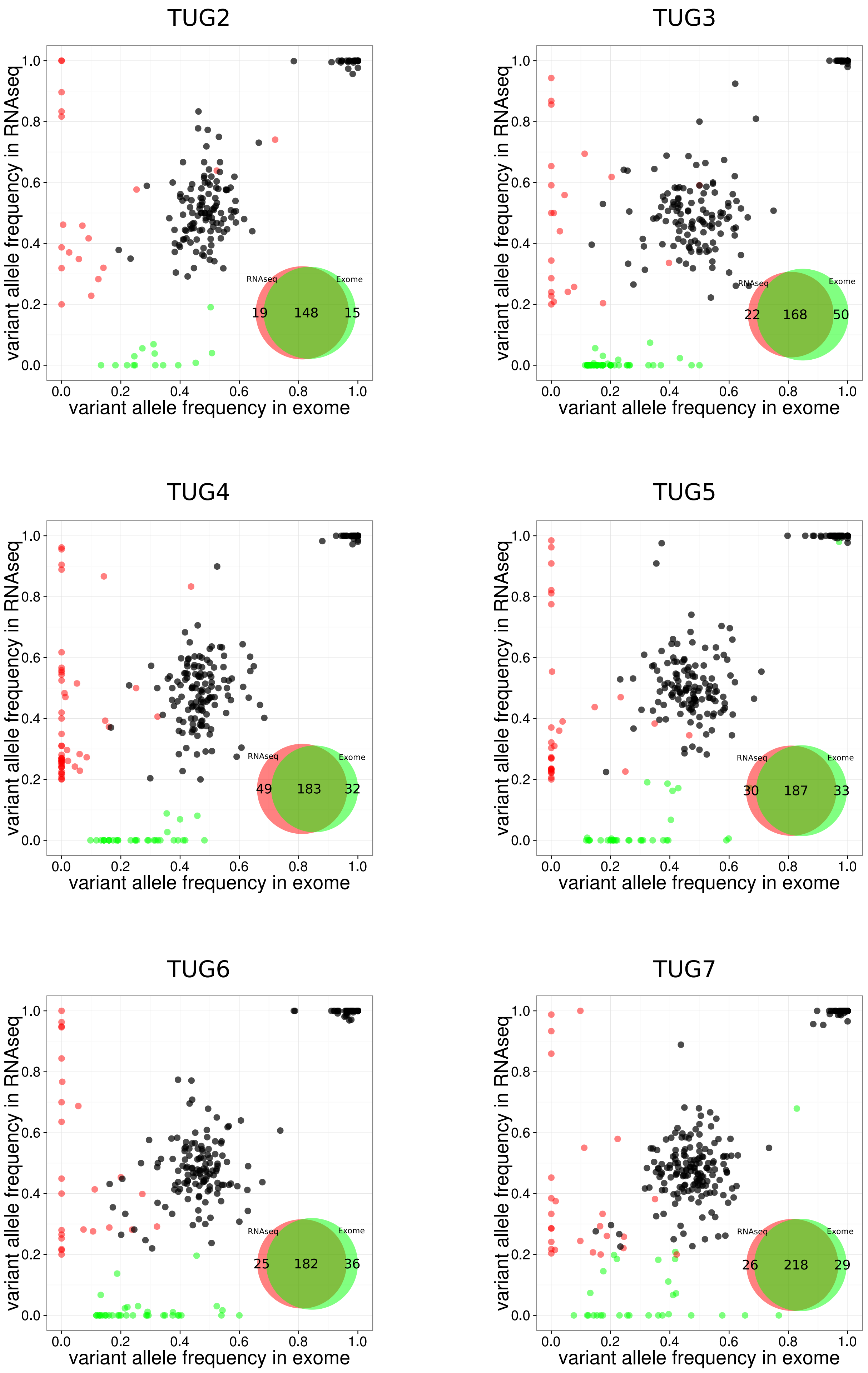

TUG2
variant allele frequency in RNAseq
variant allele frequency in exome
RNAseq
Exome
19
148
15
TUG3
variant allele frequency in RNAseq
variant allele frequency in exome
RNAseq
Exome
22
168
50
TUG4
variant allele frequency in RNAseq
variant allele frequency in exome
RNAseq
Exome
49
183
32
TUG5
variant allele frequency in RNAseq
variant allele frequency in exome
RNAseq
Exome
30
187
33
TUG6
variant allele frequency in RNAseq
variant allele frequency in exome
RNAseq
Exome
25
182
36
TUG7
variant allele frequency in RNAseq
variant allele frequency in exome
RNAseq
Exome
26
218
29
