# Supplementary material for: Comprehensive Analysis of Transcriptome Variation Uncovers Known and Novel Driver Events in T-Cell Acute Lymphoblastic Leukemia
Source: PLoS Genet. 2013 Dec 19;9(12):e1003997. doi: 10.1371/journal.pgen.1003997 (PMC3868543; doi:10.1371/journal.pgen.1003997)
Supplement: Figure S6 — INDELs in TLE92 and TLE87 are detected after mapping with a different aligner. The screenshots from UCSC genome browser shows (A) the 4 bp deletion in PTEN (note that only a part of the alignment was shown) and (B) 1 bp deletion in KDM6A. In both cases BWA transcriptome-only mapping was coupled to BLAT genome mapping. In (C) and (D), TopHat2 transcriptome-only mapping coupled with BLAT genome mapping was displayed for PTEN and KMD6A INDELs, respectively. (PDF) [file pgen.1003997.s006.pdf]

A

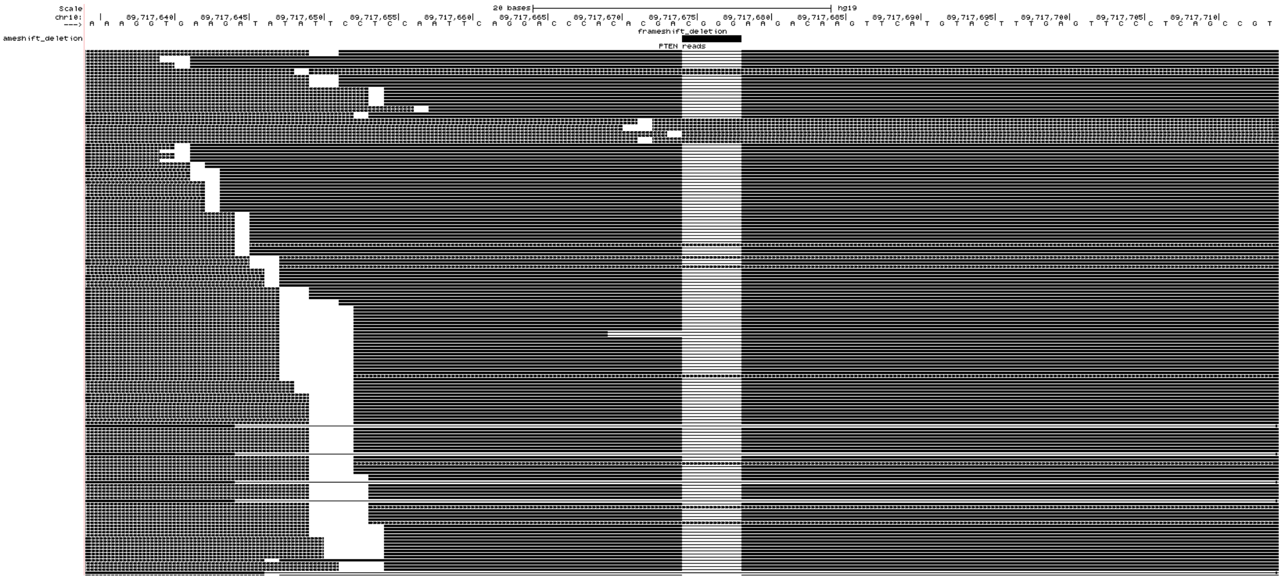
Scale
20 bases
hg19
chr10:
89,717,640
89,717,645
89,717,650
89,717,655
89,717,660
89,717,665
89,717,670
89,717,675
89,717,680
89,717,685
89,717,690
89,717,695
89,717,700
89,717,705
89,717,710
A A A G G T G A A G A T A T A T T C C T C C A A T T C A G G A C C C A C A C G A C G G G A A G A C A A G T T C A T G T A C T T T G A G T T C C C T C A G C C G T
frameshift_deletion
ameshift_deletion
PTEN reads

B

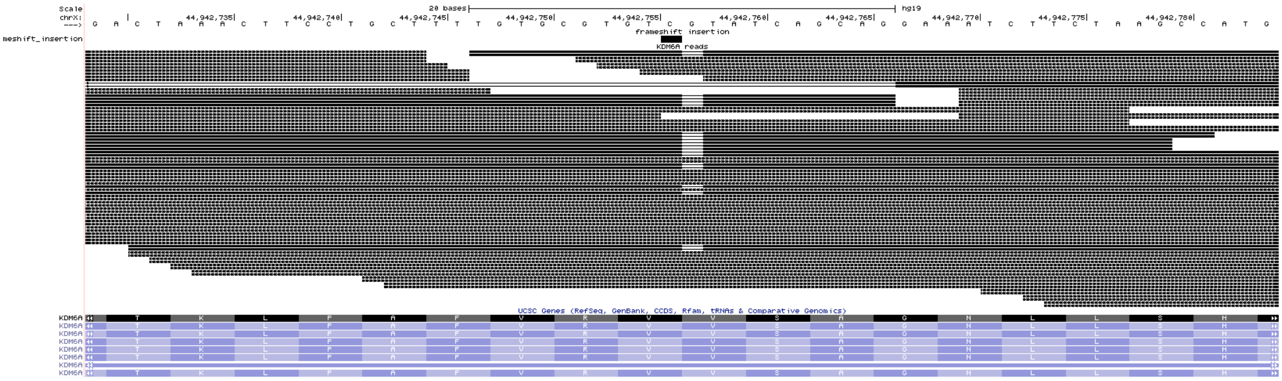
Scale
20 bases
hg19
chrX:
44,942,735
44,942,740
44,942,745
44,942,750
44,942,755
44,942,760
44,942,765
44,942,770
44,942,775
44,942,780
G A C T A A A C T T C C T G C T T T T G T G C G T G T C G T A T C A G C A G G A A A T C T T C T A A G C C A T G
frameshift insertion
meshift_insertion
KDM6A reads
UCSC Genes (RefSeq, GenBank, CCDS, Rfam, tRNAs & Comparative Genomics)
KDM6A
T K L F A F V R V V S A G N L L S H

C

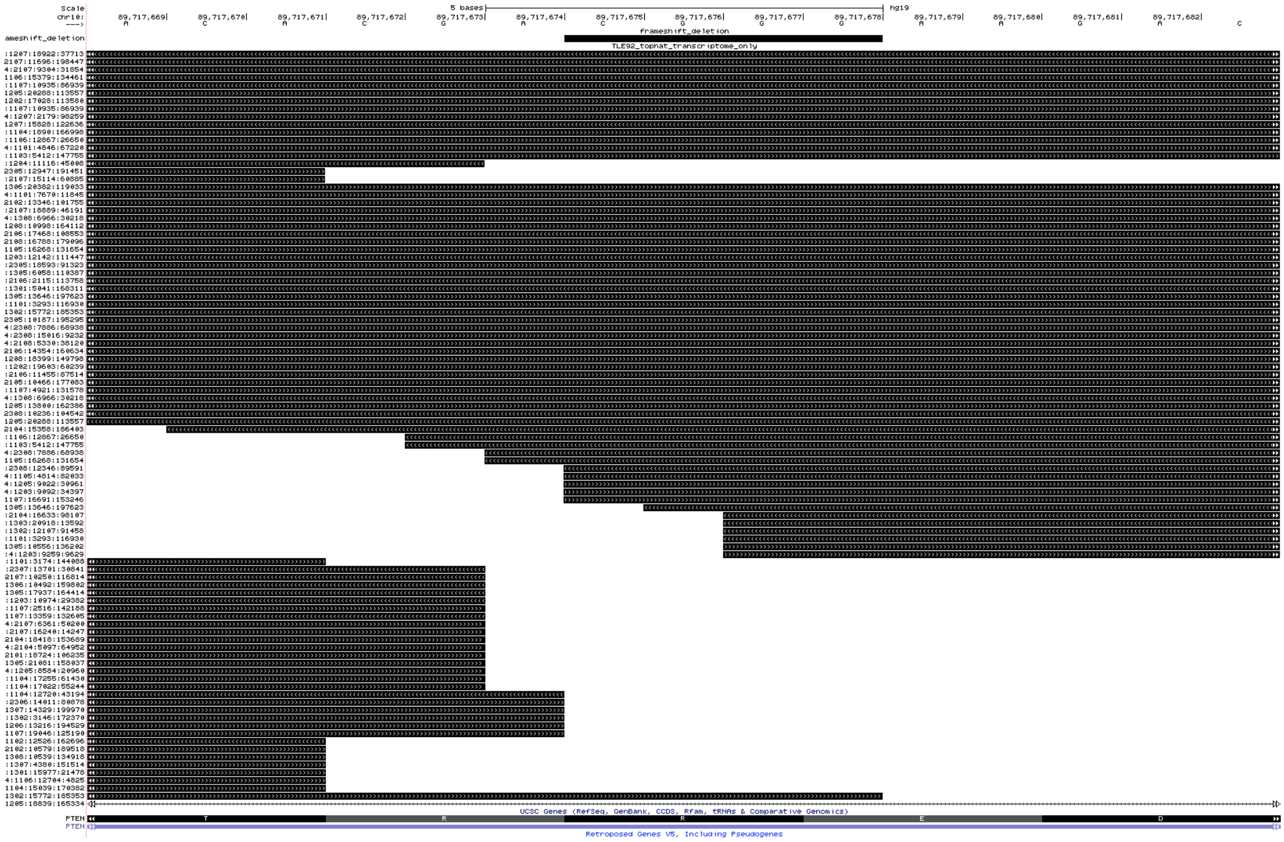

frameshift_deletion
TLE92_tophat_transcriptome_only
UCSC Genes (RefSeq, GenBank, CCDS, Rfam, tRNAs & Comparative Genomics)
PTEN
Retroposed Genes V5, Including Pseudogenes

D

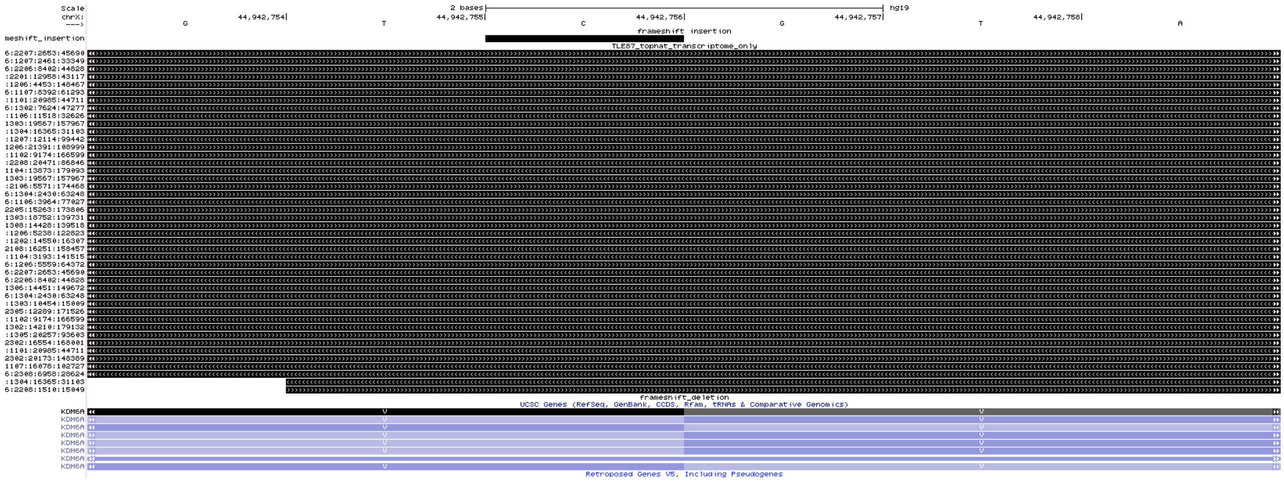

frameshift_insertion
TLE87_tophat_transcriptome_only
UCSC Genes (RefSeq, GenBank, CCDS, Rfam, tRNAs & Comparative Genomics)
KDM6A
Retroposed Genes V5, Including Pseudogenes
